# High-speed domain wall racetracks in a magnetic insulator

**DOI:** 10.1038/s41467-019-12676-7

**Published:** 2019-10-18

**Authors:** Saül Vélez, Jakob Schaab, Martin S. Wörnle, Marvin Müller, Elzbieta Gradauskaite, Pol Welter, Cameron Gutgsell, Corneliu Nistor, Christian L. Degen, Morgan Trassin, Manfred Fiebig, Pietro Gambardella

**Affiliations:** 1 Department of Materials, ETH Zurich, 8093 Zurich, Switzerland; 2 Department of Physics, ETH Zurich, 8093 Zurich, Switzerland

**Keywords:** Surfaces, interfaces and thin films, Magnetic devices, Spintronics

## Abstract

Recent reports of current-induced switching of ferrimagnetic oxides coupled to heavy metals have opened prospects for implementing magnetic insulators into electrically addressable devices. However, the configuration and dynamics of magnetic domain walls driven by electrical currents in insulating oxides remain unexplored. Here we investigate the internal structure of the domain walls in Tm_3_Fe_5_O_12_ (TmIG) and TmIG/Pt bilayers, and demonstrate their efficient manipulation by spin–orbit torques with velocities of up to 400 ms^−1^ and minimal current threshold for domain wall flow of 5 × 10^6^ A cm^−2^. Domain wall racetracks are defined by Pt current lines on continuous TmIG films, which allows for patterning the magnetic landscape of TmIG in a fast and reversible way. Scanning nitrogen-vacancy magnetometry reveals that the domain walls of TmIG thin films grown on Gd_3_Sc_2_Ga_3_O_12_ exhibit left-handed Néel chirality, changing to an intermediate Néel–Bloch configuration upon Pt deposition. These results indicate the presence of interfacial Dzyaloshinskii–Moriya interaction in magnetic garnets, opening the possibility to stabilize chiral spin textures in centrosymmetric magnetic insulators.

## Abstract (web summary)

Fast and low-power electrical control of magnetic textures is expected to enable a new generation of computational devices. Here the authors show how chiral interactions determine the structure of domain walls in Tm_3_Fe_5_O_12_ and lead to efficient current-driven wall motion.

## Introduction

Spintronics relies on the use of current-induced torques for manipulating the magnetization of thin films and nanodevices^1^. Owing to spin–orbit coupling, charge currents flowing in heavy metals, such as Pt, Ta, or W, generate spin currents that exert a torque onto an adjacent ferromagnetic layer^2,3^. These so-called spin–orbit torques (SOTs) are capable of reversing the magnetization of ferromagnets in a highly efficient and ultra-fast manner^4–8^, as well as driving domain walls (DWs) at very high velocities^9–11^. Most studies in this area, however, have been performed on ultrathin metallic ferromagnets, for which extensive characterizations of the DW structure and velocity have been reported^12–20^.

Magnetic insulators offer exciting perspectives for spintronic and magnonic applications beyond conventional metallic systems^21^. In particular, ferrimagnetic rare-earth garnets coupled to heavy metal layers have attracted attention due to the possibility of electrically exciting and detecting propagating magnons^22–25^, as well as for their low-power and high-frequency magnetization dynamics^26^. Despite increasing interest in such systems, however, the electrical manipulation of the equilibrium magnetization has not been investigated in detail. Current-induced switching of magnetic insulators has been only recently demonstrated in Tm_3_Fe_5_O_12_ (TmIG) and BaFe_12_O_19_ in combination with either Pt or W layers^27–30^. These studies relied on magnetoresistance measurements to detect the orientation of the magnetization, from which the dynamics of the switching process cannot be inferred.

In this work, we present a combined scanning nitrogen-vacancy (NV) magnetometry and spatially resolved magneto-optic Kerr effect (MOKE) study of the DW structure and dynamics driven by SOTs in racetrack structures embedded in a TmIG layer. We demonstrate highly efficient current-induced DW motion in TmIG/Pt, with mobility comparable or larger than metallic ferromagnets, a remarkable low threshold for DW flow, and very small depinning fields. We further provide a direct characterization of the DW width and internal structure in thin-film TmIG and TmIG/Pt bilayers. Previous studies in garnets were only able to provide estimates of the DW width based on indirect or diffraction-limited optical measurements, with reported values ranging from tens of nanometers to micrometers^31–34^. Scanning NV magnetometry reveals that the DWs in TmIG films are only ~20 nm wide and have a well-defined chiral structure, which changes from the left Néel in TmIG to intermediate left Néel–Bloch in TmIG/Pt. Given that the crystal structure of TmIG is centrosymmetric, these findings evidence the presence of strong interfacial Dzyaloshinskii–Moriya interaction (DMI) in TmIG grown on substituted gadolinium gallium garnet Gd_3_Sc_2_Ga_3_O_12_ (SGGG), which is attenuated by the deposition of Pt. The DMI is the key ingredient required to stabilize chiral Néel DWs in ferromagnets and ferrimagnets with perpendicular magnetization, which can then be driven by SOTs at very high velocities^10–15^. In contrast with metallic ferromagnets, TmIG thin films support the formation of Néel DWs without introducing heavy metal layers. Our results show that ferrimagnetic garnets are ideal materials for fabricating efficient and high-speed DW racetracks.

## Results

### Local switching in continuous TmIG films

**Fig. 1:**
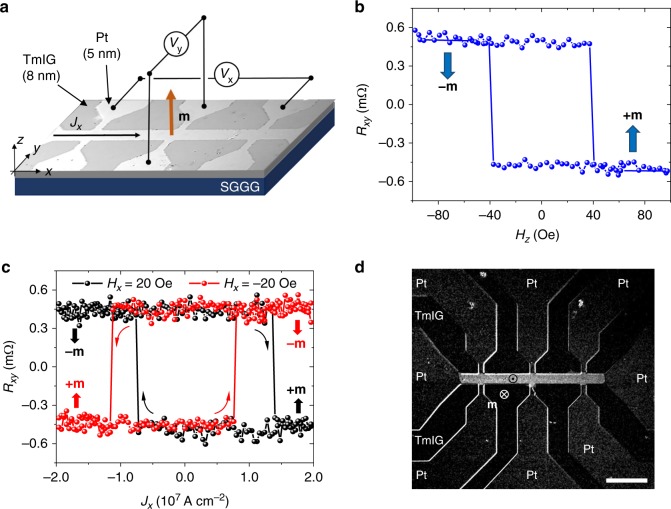
Device schematics and local switching of TmIG. **a** Optical image of a Pt Hall bar patterned on TmIG with superposed electric wiring, coordinate system, and magnetization vector **m**. **b** Hall resistance *R*_*xy*_ as a function of *H*_*z*_. The anomalous Hall-like signal arises from the interaction of the spin current generated in the Pt layer with the out-of-plane magnetization component *m*_*z*_ of TmIG^27,54^, leading to a high (low) *R*_*xy*_ for −**m** (+**m**). The data are shown after subtraction of a constant sample-dependent offset. **c** Electrical measurement of current-induced switching of TmIG (*t*_p_ = 1 ms, *H*_*x*_ = + 20, black dots, and −20 Oe, red dots). Note that, starting from a film saturated in the –**m** state, higher current densities are required to induce forward switching relative to backward switching. The same behavior is observed for ±*H*_*x*_, thus ruling out a misalignment of the sample as a possible explanation for this effect. **d** Wide-field differential MOKE image of a TmIG/Pt device after injection of a current pulse (*J*_*x*_ = 0.94 × 10^8^ A cm^−2^, *t*_p_ = 150 ns, *H*_*x*_ = 100 Oe). The film was initially saturated in the –**m** state by applying a magnetic field *H*_*z*_ = −100 Oe. The bright contrast shows that only the TmIG region underneath the Pt current line has switched to +**m**. Scale bar, 40 μm

TmIG(8 nm)/Pt(5 nm) bilayers were grown on SGGG (111)-oriented substrates by a combination of pulsed laser deposition for epitaxial growth of the garnet and in-situ dc sputtering for Pt. The numbers between parentheses indicate the thickness of each layer. Pt current lines were patterned in the shape of Hall bars by optical lithography and etching of the metal, leaving a continuous TmIG film (see Methods). Figure 1a shows an optical image of a TmIG/Pt device. The structural, topographic, magnetic, and electric characterization of the TmIG film and the TmIG/Pt bilayer are reported in the Supplementary Notes 1–3.

The magnetic state of the TmIG film underneath the Pt current line, +**m** (up) or −**m** (down), can be read electrically by measuring the transverse Hall resistance *R*_*xy*_, as shown in Fig. 1b during a sweep of the out-of-plane magnetic field *H*_*z*_. The measurement confirms that the films exhibit robust perpendicular magnetic anisotropy with a coercive field of about 40 Oe. In agreement with a previous report^27^, the magnetization of TmIG can be deterministically switched upon the application of a current pulse of sufficient current density *J*_*x*_ in the presence of a constant in-plane field *H*_*x*_ (Fig. 1c). The switching polarity is determined by the damping-like component of the SOT^2–4^, which stabilizes +**m** for *J*_*x*_ parallel to *H*_*x*_ and –**m** for *J*_*x*_ antiparallel to *H*_*x*_ in TmIG/Pt. Notably, we found that full switching can be achieved with pulses of 1 ms at current densities below 10^7^ A cm^−2^ for an in-plane field as small as |*H*_*x*_| = 20 Oe, which confirms the high quality of our devices (see also Supplementary Note 3).

A distinctive feature of our experiments is that TmIG covers the entire substrate, but switching occurs only in the region defined by the Pt current line. This is clearly seen in Fig. 1d, which shows a differential MOKE image of a TmIG/Pt device after the application of a current pulse. The bright contrast coinciding with the Pt current line shows the region where the magnetization has switched from −**m** to +**m**, demonstrating that it is possible to control the magnetization of a continuous TmIG film in a local way without altering the magnetic moments of the surroundings. Only in the presence of an out-of-plane field, or for a significant Oersted field and Joule heating produced by an intense current pulse, the switched magnetic domain may extend beyond the Pt line (see Supplementary Note 4). Such local control of the magnetization is unique to magnetic insulators due to the confinement of the current in the metal overlayer. As discussed further below, we also find that the surrounding magnetic medium influences the switching dynamics underneath the Pt current line. This is seen by the fact that a larger current is required for inducing down-to-up switching relative to up-to-down switching when starting from a homogenously magnetized TmIG film pointing down (Fig. 1c).

### Chiral DWs in TmIG revealed by scanning NV magnetometry

**Fig. 2:**
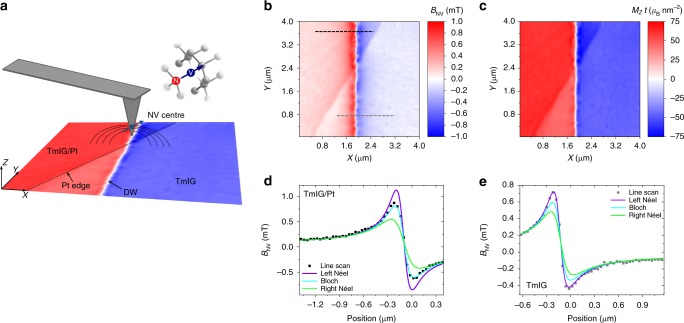
Domain wall structure and chirality in TmIG and TmIG/Pt measured by scanning NV magnetometry. **a** Schematic of the NV probe and sample. The color code represents the out-of-plane component of the surface magnetization of the film, with red (blue) corresponding to areas of opposite magnetization. A DW crosses the TmIG/Pt and TmIG regions. The inset shows a NV center within the diamond lattice and the corresponding spin quantization axis. **b** Stray field *B*_NV_(*X*,*Y*) measured by scanning the diamond tip over the sample surface shown in **a**. **c** Reconstructed out-of-plane magnetic surface map *M*_*Z*_(*X*,*Y*)*t* from the data shown in **b**, where *t* is the thickness of the TmIG layer (see Methods) and *μ*_B_ the Bohr magneton. The difference in the measured surface magnetization *M*_*Z*_*t* = (66.8 ± 1.5) *μ*_B_ nm^−2^ in TmIG/Pt and *M*_*Z*_*t* = (39.5 ± 2.2) *μ*_B_ nm^−2^ in TmIG is attributed to both the etching process and to proximity-induced polarization of the Pt layer (see Supplementary Notes 2 and 7). **d**, **e** Line scans of *B*_NV_ along the dashed lines indicated in **b** (square dots). The solid lines are calculated *B*_NV_ profiles using Eqs. (1–3) assuming pure Bloch (*ψ* = 90°, cyan), left-handed Néel (*ψ* = 180°, violet), and right-handed Néel (*ψ* = 0°, green) DW structures for comparison with the measured stray field profiles. *M*_s_*t* and Δ_DW_ are set from the fits of the *B*_NV_(*X*,*Y*) map. The fits give *ψ* = (116 ± 33)° and ${\mathrm{\Delta }}_{{\mathrm{DW}}} = (17 \pm 17)$ nm for TmIG/Pt, and *ψ* = (173 ± 17)° and ${\mathrm{\Delta }}_{DW} = (27 \pm 6)$ nm for TmIG

As SOT-induced switching is strongly dependent on the DW structure^12–15^, we use scanning NV magnetometry to reveal the DW magnetization profile in both TmIG and TmIG/Pt layers. The technique is based on a single NV defect located at the apex of a diamond tip, which senses the magnetic stray field *B*_NV_(*X*,*Y*) emanating from a magnetic surface with high spatial resolution (Fig. 2a)^17,35,36^. Figure 2b shows *B*_NV_(*X*,*Y*) of the TmIG film measured in a region where a DW intersects an area partially covered by Pt. From this measurement, we reconstruct the out-of-plane component of the surface magnetization *M*_*Z*_(*X*,*Y*)*t* (see Methods), as shown in Fig. 2c. Although the DW runs continuously across the Pt edge, the line scans of *B*_NV_(*X*,*Y*) shown in Fig. 2d, e reveal that the DW structure changes going from TmIG/Pt to TmIG. In order to extract the magnetization profile of the DW from these measurements, we fit *B*_NV_(*X*,*Y*) by assuming that the magnetization components in the rotated coordinate system *XYZ* (Fig. 2a) vary as^17,33^

1 $$\begin{array}{*{20}{l}} {M_X\left( X \right)} \hfill & = \hfill & {M_{\mathrm{s}}\frac{{{\mathrm{cos}}\psi }}{{{\mathrm{cosh}}\left( {\frac{X}{{{\mathrm{\Delta }}_{{\mathrm{DW}}}}}} \right)}},} \hfill \\ {M_Y\left( X \right)} \hfill & = \hfill & {0,} \hfill \\ {M_Z\left( X \right)} \hfill & = \hfill & { - M_{\mathrm{s}}{\mathrm{tanh}}\left( {\frac{X}{{{\mathrm{\Delta }}_{{\mathrm{DW}}}}}} \right),} \hfill \end{array}$$

where *ψ* defines the angle of the in-plane magnetization direction with respect to the *X*-axis, Δ_DW_ is the DW width, and *M*_s_ the saturation magnetization. Figure 2d, e compares representative *B*_NV_(*X*,*Y*) line profiles for TmIG/Pt and TmIG together with the stray field profile of a pure Bloch wall (*ψ* = 90°), a left Néel wall (*ψ* = 180°), and a right Néel wall (*ψ* = 0°). The best fits of the *B*_NV_(*X*,*Y*) line profiles give *ψ* = (116 ± 33)° and *ψ* = (173 ± 17)°, corresponding to an intermediate left-handed Néel–Bloch wall for TmIG/Pt and a left-handed Néel wall for TmIG, respectively. The DW widths are Δ_DW_ = (17 ± 17) nm and Δ_DW_ = (27 ± 6) nm for TmIG/Pt and TmIG, respectively (see Methods). Despite the large uncertainty in Δ_DW_, which is due to the weak dependence of *B*_NV_ on Δ_DW_, and which prevents us to determine the relative change of DW width between TmIG/Pt and TmIG, the fits show that the DWs in 8 nm-thick TmIG are very narrow. Measurements performed in a reference unetched TmIG layer of the same thickness showed that *ψ* = 180° and Δ_DW_ = (20 ± 4) nm (see Supplementary Note 7), which confirms the left-handed Néel chirality and the narrow width of the DWs in thin TmIG films grown on SGGG.

The change of the DW chirality from left-handed Néel to an intermediate left-handed Néel–Bloch configuration in going from TmIG to TmIG/Pt is a compelling indication of the presence of negative DMI in the bare TmIG layer, most likely due to symmetry breaking at the SGGG/TmIG interface. The deposition of Pt reduces the DMI, which we ascribe to the presence of positive DMI at the TmIG/Pt interface, consistently with the sign of the DMI found in metallic ferromagnetic/Pt bilayers^14,15,17^. These findings have important consequences for the operation of DW racetracks in magnetic insulators, because the reduced Δ_DW_ favors the localization of DWs, whereas the finite DMI allows for their efficient manipulation by SOTs.

### Spatially resolved switching dynamics

**Fig. 3:**
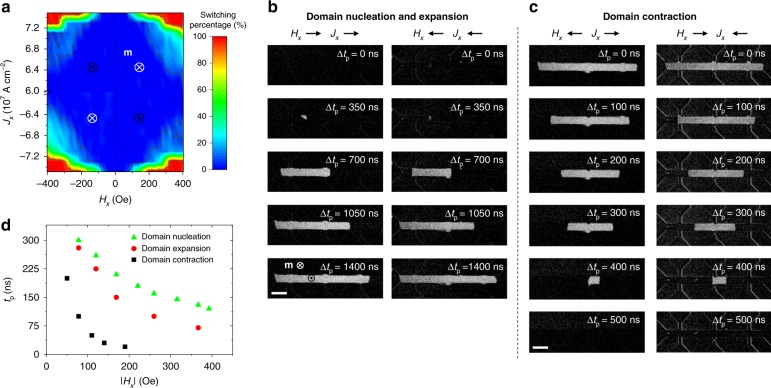
Current-induced DW dynamics in embedded TmIG racetracks. **a** Magnetization switching diagram showing the percent change of the magnetization underneath the Pt current line as a function of *J*_*x*_ and *H*_*x*_. For each switching event, the initial **m** was always set fully up or down by applying an out-of-plane field *H*_*z*_ = + 100 or −100 Oe, respectively (+**m** for ±*H*_*x*_, $\mp J_x$ and –**m** for ±*H*_*z*_, ±*J*_*z*_ according to the symmetry of SOT switching). Forward switching is triggered by a single current pulse with *t*_p_ = 500 ns. **b** Sequence of differential MOKE images showing current-induced domain nucleation and expansion and **c** domain contraction for the different combinations of *H*_*x*_ and *J*_*x*_ that allow each scenario to take place. Δ*t*_p_ = *Nt*_p_ is the accumulated pulse time after applying *N* current pulses of length *t*_p_. The pulse length is *t*_p_ = 350 and 50 ns, and the current density |*J*_*x*_| = 0.65 × 10^8^ and 0.75 × 10^8^ A cm^−2^ for **b** and **c**, respectively. |*H*_*x*_| = 250 Oe in all cases. Bright and dark MOKE contrasts correspond to +**m** and –**m** states, respectively. It is noteworthy that the TmIG film not covered by Pt remains fully down magnetized. Scale bars, 20 μm **b**, **c**. **d** Switching diagram showing the minimum pulse length *t*_p_ required to nucleate a domain starting from a fully saturated film (green triangles), to expand a domain (red circles), and to contract a domain (black squares) as a function of |*H*_*x*_|. The current density of the pulses is fixed to |*J*_*x*_| = 0.75 × 10^8^ A cm^−2^. The results show that it is easier to move a DW than to nucleate a domain, and that the onset of DW motion is lower for contraction relative to expansion

In order to prove this last point, we investigate the switching dynamics and current-induced DW motion below the Pt line. We refer to the switching of the magnetization starting from a homogeneously magnetized TmIG layer as forward switching (domain nucleation and expansion), and to the return to a homogenous magnetic state starting from a reversed domain as backward switching (domain contraction). Figure 3a shows the relative change in the magnetization induced by a single forward switching current pulse as a function of *H*_*x*_ and *J*_*x*_. Within the experimental error, we find that the switching diagram is symmetric upon inversion of *H*_*x*_ or *J*_*x*_, indicating that the SOT efficiency is the same for up-to-down and down-to-up switching and independent on the current direction. Distinct to electrical reading^27–29^, which is only sensitive to the magnetic moments in the vicinity of the Hall cross (Fig. 1b, c and Supplementary Note 5), MOKE measurements reveal that for a wide range of *H*_*x*_ and *J*_*x*_ only partial switching is achieved. We thus study the distribution and evolution of reversed magnetic domains induced by a sequence of current pulses. As we expect an influence of the surrounding TmIG on the magnetization dynamics underneath the Pt current line (Fig. 1c) and because the switching process is symmetric upon inverting both **m** and **H**^4,8^, we investigate the forward and backward switching processes for one fixed initial state of the film (−**m**).

Figure 3b, c shows two representative sequences of differential MOKE images taken during forward switching and backward switching, respectively. For each case, we compare the combinations of field and current that allow for domain nucleation and expansion (±*H*_*x*_, ±*J*_*x*_) and domain contraction (±*H*_*x*_, $\mp J_x$). These images reveal that forward switching occurs via nucleation of a reversed domain at a defect site (as confirmed by a series of repetitions) and subsequent domain expansion along the Pt current line, with comparable speeds for both DWs on the left- and right-hand sides of the domain. Backward switching takes place by pushing the outer DWs towards the center of the domain. Similar dynamics—for either nucleation and expansion or contraction—is observed upon inverting *H*_*x*_ and *J*_*x*_. The different timescales of the switching processes (see Fig. 3b, c) indicate that domain contraction is significantly faster than domain expansion. We attribute this asymmetry to the tendency of the reversed domain to shrink in order to reduce the DW surface tension^37,38^. The latter is proportional to the DW length and can thus significantly offset the balance of SOT, pinning potential, and demagnetizing field. Accordingly, we find that the minimum pulse length required to induce DW motion upon contraction is much smaller than for expansion (Fig. 3d).

### DW velocity and DMI

**Fig. 4:**
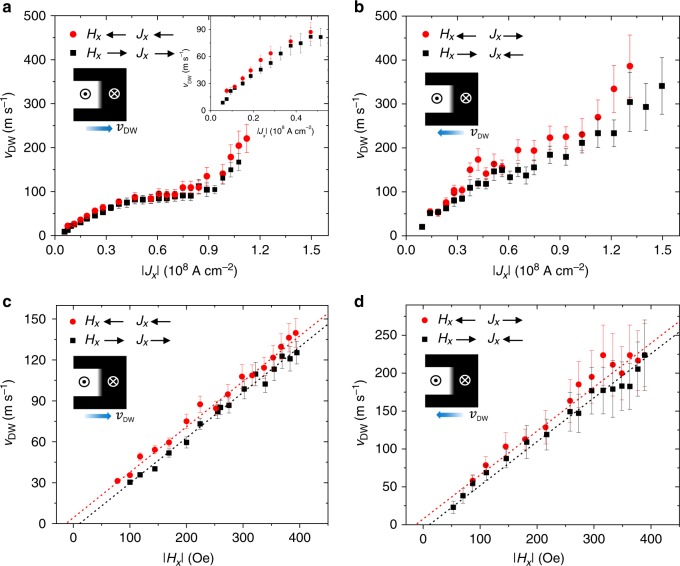
DW velocity upon expansion and contraction of domains in TmIG/Pt surrounded by uniformly magnetized TmIG. **a** *v*_DW_ of an up–down DW (corresponding to a +**m** domain in a –**m** medium) as a function of *J*_*x*_ upon expansion and **b** contraction. The magnetic field is set to |*H*_*x*_| = 300 Oe. The inset in **a** shows an enlarged view of *v*_DW_ in the flow regime at low current density. **c** *v*_DW_ of an up–down DW as a function of *H*_*x*_ upon expansion and **d** contraction. The current density is fixed at |*J*_*x*_| = 0.75 × 10^8^ A cm^−2^ in either case. DWs move faster when *J*_*x*_ is opposite to *v*_DW_ (red symbols), indicating the presence of a weak DMI favoring left-handed Néel chiral DWs. Dashed lines in either **c** or **d** are linear fits to the experimental data assuming the same slope of *v*_DW_(*H*_*x*_) for both polarities of *J*_*x*_ and opposite magnetic field at which *v*_DW_ crosses zero, which allows evaluating the effective DMI field |*H*_DMI_| ~ 12 ± 3 Oe (see ref. ^15^). The error bars account for the uncertainty in estimating *v*_DW_ from each measurement

Measurements of the DW velocity *v*_DW_ are reported in Fig. 4 for an up–down DW as a function of *J*_*x*_ and *H*_*x*_ during both domain expansion and domain contraction. *v*_DW_ is evaluated by considering the total DW displacement after a sequence of current pulses of length *t*_p_ as the DW moves along the Pt current line. As the same *t*_p_ does not allow for sampling a large *H*_*x*_, *J*_*x*_ parameter space, we used longer (shorter) pulses for smaller (larger) *H*_*x*_, *J*_*x*_ values. It is noteworthy that, up to |*J*_*x*_| ≲ 1 × 10^8^ A cm^−2^, the DW velocity remains almost constant when changing *t*_p_, indicating that it is not influenced by inertia, and that Joule heating plays a minor role on the DW velocity in this regime (see Methods and Supplementary Fig. 11). Our measurements reveal robust DW velocities of up to ~200 m s^−1^ for domain expansion (Fig. 4a) and ~400 m s^−1^ for domain contraction (Fig. 4b), which are comparable to the ones found in all-metallic structures under similar conditions^9,14,15^. Most remarkably, however, the DW mobility $\mu _{{\mathrm{DW}}} = \frac{{v_{{\mathrm{DW}}}}}{{J_x}}$ reaches values in excess of 3 × 10^−10^ m^3^ A^−1^ s^−1^ for *J*_*x*_ = 5 × 10^7^ A cm^−2^, which is comparable to that observed in compensated metallic ferrimagnets^11,39^. In contrast, most metallic ferromagnets feature *μ*_DW_ = 0 in this current range^9,10,14,15^.

The linear increase of *v*_DW_ with *J*_*x*_ for expanding and contracting walls (Fig. 4a, b) further reveals a very low onset of the DW flow regime (≲5 × 10^6^ A cm^−2^) compared with conventional ferromagnetic layers^9,10,14,15^. This behaviour is attributed to the reduced depinning field of TmIG, **~**1–2 Oe (see Supplementary Fig. 6), which is one to two orders of magnitude smaller than in metallic and semiconducting thin-film ferromagnets with perpendicular magnetic anisotropy^40^. Upon increasing the current, *v*_DW_ reaches a plateau between **~**0.5 × 10^8^ A cm^−2^ and **~**0.9 × 10^8^ A cm^−2^, followed by a further upturn. The plateau indicates the saturation of the DW velocity at $v_{{\mathrm{DW}}}^{{\mathrm{sat}}} \approx \gamma \Delta _{{\mathrm{DW}}}\frac{\pi }{2}(H_x + H_{{\mathrm{DMI}}})$, which occurs at a current density $J_x > > \frac{{2e\alpha \mu _0M_{\mathrm{s}}t}}{{\hbar \theta _{{\mathrm{SH}}}}}(H_x + H_{{\mathrm{DMI}}})$, where *γ* is the gyromagnetic ratio, *α* the damping constant, *e* the electron charge, *ħ* the reduced Planck constant, *μ*_0_ the vacuum permeability, *θ*_SH_ the effective spin Hall angle of Pt, and *H*_DMI_ the effective DMI field^12,16^. Taking *γ* ~ 1.43 × 10^7^ Oe^−1^ s^−1^ (ref. ^41^) and Δ_DW_ ~ 20 nm (Fig. 2), and by approximating *H*_*x*_ + *H*_DMI_ ≈ *H*_*x*_ = 300 Oe, we find that $v_{{\mathrm{DW}}}^{{\mathrm{sat}}}\sim 135\,{\mathrm{m}}\,{\mathrm{s}}^{ - 1}$, which agrees well with the experimental data (Fig. 4a, b). It is noteworthy that the influence of the surrounding film on *v*_DW_ is not considered in this estimate. In fact, the DW surface tension results in a variation of the DW mobility between contracting and expanding domains by approximately a factor of 1.75, a difference that remains constant as a function of field and current for DWs moving in the flow and saturation regimes up to |*J*_*x*_| ≲ 0.9 × 10^8^ A cm^−2^ (see Supplementary Fig. 12). The further increase of *v*_DW_ beyond saturation, which is typical also of metallic ferromagnets^10,15^, is attributed to the influence of Joule heating and Oersted field (see Supplementary Note 4 and Supplementary Figs. 11 and 12). In this high current regime (|*J*_*x*_| ≲ 0.9 × 10^8^ A cm^−2^), the steeper slope of *v*_DW_(*J*_*x*_) for domain expansion relative to domain contraction is consistent with the longer (shorter) pulses employed for expanding (contracting) domains (see Fig. 3d), resulting in a larger (smaller) Joule heating.

In agreement with the presence of DMI inferred from the DW magnetization profile, we observe a slightly larger *v*_DW_ when the DW moves against the direction of the current (red symbols in Fig. 4). The same behaviour is also confirmed for down–up DWs (see Supplementary Fig. 13). This asymmetry, which is characteristic of chiral Néel DWs^14,15^, is consistent with the partially left-handed Néel chirality derived from scanning NV magnetometry and the sign of the torques in TmIG/Pt (see Supplementary Fig. 14 for more details). Provided that the dynamics of the DWs is restricted to the flow regime, we can estimate the effective internal DMI field of the DWs by fitting *v*_DW_(*H*_*x*_) to a linear function and extrapolating it to *v*_DW_ = 0 (see ref. ^15^). The fit yields |*H*_DMI_| ~ 12 ± 3 Oe (Fig. 4c, d), which allows us to calculate the effective DMI constant as^9,14^ $D = \mu _0H_{{\mathrm{DMI}}}M_{\mathrm{s}}{\mathrm{\Delta }}_{{\mathrm{DW}}}\sim - 2 \pm 2$ μJ m^−2^, where we have taken *M*_s_ = (6.0 ± 1.0) × 10^4^ A m^−1^ and Δ_DW_ ~ 20 nm. Alternatively, the DMI constant can be estimated from the DW chirality using the equation cos *ψ* = *D*/*D*_c_, where $D_{\mathrm{c}} = 2\mu _0M_{\mathrm{s}}^2t\ln 2/\pi ^2$ (see refs. ^42,43^). This estimate gives *D* ~ −2.3 ± 2.6 μJ m^−2^, in good agreement with the value obtained from the analysis of the DW velocity. From the NV measurements of the bare TmIG, we estimate that the DMI of SGGG/TmIG is *D* ~ −5.3 ± 1.8 μJ m^−2^.

The DMI in TmIG is thus two to three orders of magnitude smaller compared with ultrathin metallic ferromagnet/Pt bilayers^14,15,18^ and one to two orders of magnitude smaller than that of ferrimagnetic metal/Pt bilayers^11^. As the DW mobility in the flow regime is proportional to Δ_DW_/*αM*_s_, the large mobility and high DW velocities in TmIG/Pt appear as the direct consequence of the small *M*_s_ and low-damping *α* typical of garnet layers^31,44^. By tuning the interfacial DMI, we anticipate that even larger *v*_DW_ may be reached at a relatively low current density.

## Discussion

Our results demonstrate fast current-driven DW motion in a magnetic insulator and reveal the internal DW structure of thin garnet layers. The chiral Néel structure of the DWs in TmIG indicates that oxide interfaces support a finite DMI even in the absence of heavy metal layers, which makes it possible, in principle, to stabilize nontrivial topological configurations in centrosymmetric insulating magnetic thin films, such as spin spirals and skyrmions. The low current threshold for DW flow and the large DW mobility, combined with the possibility of defining DW racetracks embedded in a continuous magnetic medium, make TmIG extremely attractive for spintronic applications. Local control of the magnetization is unique to magnetic insulators, which opens the possibility of printing arbitrary circuit paths enabling, for instance, the implementation and in-situ reconfiguration of synthetic magnetic structures with tailored magnonic bands^21,45^ and nanomagnonic waveguides^46,47^. Finally, we note that recent reports also demonstrate fast current-induced DW motion in TmIG/Pt at zero field^48^ as well as the emergence of a finite topological Hall effect above room temperature^49^. These works further prove the potential of hybrid magnetic insulator/metal heterostructures for stabilizing and manipulating chiral magnetic textures by proximity charge currents.

## Methods

### Films growth and devices fabrication

The TmIG thin films were grown by pulsed laser deposition on (111)-oriented Gd_3_Sc_2_Ga_3_O_12_ substrates (lattice constant *a* = 12.56 Å) to achieve high tensile strain (~2%), which promotes perpendicular magnetic anisotropy^50^. The substrate temperature was 650 °C, the oxygen pressure was 0.2 mbar, whereas the laser fluence and repetition rate were set to 1.35 J cm^−2^ and 8 Hz, respectively. After deposition, the samples were cooled in 200 mbar oxygen at a rate of −10 K/min. To ensure a high quality of the TmIG/Pt interface, the TmIG films were directly transferred to the sputter chamber without breaking vacuum, where the Pt layer was deposited at room temperature for 3 min at a power of 10 W in 0.05 mbar Ar. The thickness of the layers was calibrated by X-ray reflectometry. For the sample presented in the main text, the thicknesses of TmIG and Pt were 8.3 and 5.0 nm, respectively. Atomic force microscopy measurements of the surface topography showed a root-mean-square roughness of about 0.15 nm over a ~5 × 5 µm^2^ area (see Supplementary Fig. 2). The films were magnetically characterized in a superconducting quantum interference vibration sample magnetometer system. The Pt layer was patterned into Hall bars (consisting of three Hall crosses separated by *L* = 50 µm with a total channel length of 140 µm and width *W* = 10 µm) by photolithography and subsequent Argon plasma etching. According to the topographic and magnetic characterization of the patterned TmIG/Pt and reference TmIG samples, we estimate that etching of Pt results also in partial etching and passivation of TmIG, leading to a reduction of the effective thickness of TmIG in the etched region by ~1 nm (see Supplementary Notes 2 and 3).

### Electric transport measurements

The longitudinal and transverse Hall resistances *R*_*xx*_ = *V*_*x*_/*I*_*x*_ and *R*_*xy*_ = *V*_*y*_/*I*_*x*_, respectively, were measured by applying an alternating current of amplitude *I*_*x*_ = 0.3 mA and frequency *f* = 11 Hz, and by recording the first harmonic longitudinal (*V*_*x*_) and transverse (*V*_*y*_) voltages, as shown schematically in Fig. 1a.

### MOKE measurements

We used a home-built wide-field polar MOKE microscope with Koehler illumination to measure the out-of-plane component of TmIG. As a light source, we employed a collimated light-emitting diode from Prizmatix, Ltd, model MIC-LED-455L, whose spectral emission is characterized by a maximum peak emission at 454 nm, centroid at 455 nm, and a full width at half maximum of 28 nm. Magnetic contrast was enhanced by taking differential MOKE images, i.e., each image was subtracted by a reference image captured in a fully magnetized state. The setup was equipped with two sets of orthogonal coils for the generation of out-of-plane and in-plane magnetic fields. For the switching and DW velocity studies, current pulses were injected using an AGILENT 8114A (100V/2A) pulse generator with a 50 Ω output impedance. The impedance matching with the Pt current line was achieved by connecting a 50 Ω resistance in parallel to the current line.

The relative change in the magnetization shown in Fig. 3a was evaluated by integrating the differential MOKE signal along the Pt current line (corresponding to the bright area in Fig. 1d) after the application of a single current pulse starting from a fully magnetized state.

For the domain expansion measurements (Fig. 4a, c), an initial domain was nucleated by a single current pulse at a defect site near the center of the Hall bar. For the domain contraction experiments (Fig. 4b, d), the initial domain was generated by switching the area underneath the Pt current line with a single current pulse of *t*_p_ = 150 ns, |*J*_*x*_| = 0.94 × 10^8^ A cm^−2^ and |*H*_*x*_| = 125 Oe, leading to a domain as the one shown in Fig. 1d.

In order to compare the DW velocities obtained for domain expansion and domain contraction, we studied the same DW moving back and forth over the same area. The case presented in Fig. 4 corresponds to an up–down DW moving between the center and the right end of the Hall bar. The DW velocity *v*_DW_ was evaluated by measuring the total DW displacement Δ*x* (as identified by direct MOKE imaging) obtained after the application of a series of *N*_p_ current pulses of width *t*_p_, yielding $v_{{\mathrm{DW}}} = {\mathrm{\Delta }}x/(N_{\mathrm{p}}\,t_{\mathrm{p}})$. The pulses were applied at a frequency of 1 Hz, to minimize the heat load during the experiment. *v*_DW_ was found to be nearly independent of *t*_p_—only showing a slight increase of 10% or less when doubling the pulse length, which we attribute to Joule heating—indicating that the DW motion coincides with the pulse duration (see Supplementary Fig. 11 for more details).

### Scanning NV magnetometry

Spatially resolved scans of the magnetic stray field produced by a DW in TmIG (see Fig. 2a) were acquired on a home-built nanoscale scanning diamond magnetometer (NSDM) microscope. Experiments were carried out in ambient environment and at zero magnetic bias field. The NSDM employed a monolithic diamond probe tip with a single NV center implanted at the apex (QZabre LLC, www.qzabre.com). The NV center spin resonance was monitored by optically detected magnetic resonance (ODMR) spectroscopy^36,51^ using a nearby microwave antenna (~2.9 GHz) for spin excitation and fluorescence microscopy (532 nm excitation, 630–800 nm detection) for spin state readout. The laser power employed in our measurements was 145 μW and 10 μW for the sample of the main text and the TmIG (8.5 nm) reference sample, respectively. No influence of the illumination power on the DW structure or position was noticed over time.

To convert the spin resonance frequencies to units of magnetic field, we fitted the ODMR spectrum to a double Lorentzian and extracted the frequency difference Δ*f* between the resonance peaks. The detected field *B*_NV_ is then given by $|B_{{\mathrm{NV}}}| = \frac{{{\mathrm{\pi }}\,{\mathrm{\Delta }}f}}{\gamma }$, where *γ* = 2π · 28.0 GHz/T is the electron gyromagnetic ratio. To re-establish the relative sign of *B*_NV_, we inverted (*B*_NV_ → −*B*_NV_) the image on one side of the DW (Fig. 2b). It is noteworthy that scanning NV magnetometry provides a vector projection of the magnetic field,

$$B_{{\mathrm{NV}}} = {\mathbf{B}} \cdot {\mathbf{e}}_{{\mathrm{NV}}} = {\mathrm{sin}}\theta _{{\mathrm{NV}}}{\mathrm{cos}}\phi _{{\mathrm{NV}}}B_x + {\mathrm{sin}}\theta _{{\mathrm{NV}}}{\mathrm{sin}}\phi _{{\mathrm{NV}}}{\mathrm{B}}_y + {\mathrm{cos}}\theta _{{\mathrm{NV}}}B_z,$$

because the NV center is sensitive only to fields that are parallel to its symmetry axis **e**_NV_. Here, *B* = (*B*_*x*_, *B*_*y*_, *B*_*z*_) is the vector field at the position of the NV center, and *θ*_NV_ and *ϕ*_NV_ are the polar and azimuth angles of **e**_NV_ in the laboratory frame (see Supplementary Fig. 15). The direction **e**_NV_ is determined by the crystallographic orientation of the diamond tip and the probe arrangement in the setup (see Fig. 2a). *θ*_NV_ and *ϕ*_NV_ were calibrated by a series of ODMR measurements and confirmed by line scans. For the experiments presented in Fig. 2, *θ*_NV_ = (55 ± 2)° and *ϕ*_NV_ = (83 ± 3)°.

We investigated the magnetization, spin structure, and width of the DW by analyzing the local field image *B*_NV_(*X*,*Y*) shown in Fig. 2b. In a first step, we fitted line cuts across the TmIG to TmIG/Pt step edge to extract the NV center stand-off distance, *d* = (104 ± 5) nm (see Supplementary Note 7). To characterize the chirality and width of the DW, we took line cuts of *B*_NV_ perpendicular to the DW as shown in Fig. 2b and compared them with the analytical model given through Eq. (1). The associated magnetic stray field was obtained by forward propagation of Eq. (1) in *k*-space according to^52,53^,

3 $$\begin{array}{l}\hat B_X = \hat g\left( { - k\widehat{M}_X + ik_X\widehat{M}_Z} \right)\\ \hat B_Y = \hat g\left( { - k\widehat{M}_Y + ik_Y\widehat{M}_Z} \right)\\ \hskip 32pt\hat B_Z = \hat g\left( {ik_X\widehat{M}_X + ik_Y\widehat{M}_Y + k\widehat{M}_Z} \right)\end{array}$$

where hat symbols indicate Fourier transforms, *k*_*X*_, *k*_*Y*_, and *k* = (*k*_*X*_^2^ + *k*_*Y*_^2^)^1/2^ are the in-plane *k*-space vectors, $\hat g = \frac{{\mu _0t}}{2}\left( {\frac{{1 - e^{ - kt}}}{{kt}}} \right)e^{ - kd}$ is the Fourier transform of the Green’s function^53^, and *t* is the TmIG film thickness, which is taken to be 8.3 and 7.3 nm for the TmIG/Pt and TmIG regions, respectively (see Supplementary Notes 2 and 3). It is worth noting that Eq. (3) shows that only changes in *M*_*Z*_ lead to a stray field, which is otherwise zero for a uniformly magnetized magnetic surface. The stray field is therefore strongest near the DW and decays to zero as one moves away from the DW. When taking line cuts along *X* across a DW extending along *Y*, the *Y* and $\hat M_Y$ terms become zero and Eq. (3) simplifies to $\hat B_X = \hat g\left( { - k\hat M_X + ik_X\hat M_Z} \right)$, $\hat B_Y = 0$, and $\hat B_Z = \hat g\left( {ik_X\hat M_X + k\hat M_Z} \right)$.

To extract values for *M*_s_, *ψ*, and Δ_DW_, we fitted the experimentally measured *B*_NV_ to the analytical prediction by Eqs. (1–3), with the DW position *x* = *x*_0_ as an additional fit parameter. By repeating the fitting procedure for a series of line scans, we obtained distributions for all parameters together with their means and standard deviations. A detailed description of the fitting procedure and error analysis is given in the Supplementary Note 7. It is noteworthy that we can infer Δ_DW_ values that are below the NV-to-sample distance due to the large signal-to-noise ratio in our experiments and because the stray field extends far beyond the nominal DW width (see Eq. (1)).

By assuming that the magnetization is predominantly out-of-plane, we further reconstructed the surface magnetization from the magnetic field map *B*_NV_(*X*,*Y*) by using

4 $$\hat M_Z = \frac{{ - \hat B_{{\mathrm{NV}}}\zeta}}{{g(ik_Xe_X + ik_Ye_Y - ke_Z)}},$$

where *ζ* is a low-pass filter (cutoff *λ* = *d*) that suppressed high spatial frequencies in the image^53^. Independently of the thickness of the film, Eq. (4) yields the surface magnetization of the film in units of magnetic moment/area, i.e., it provides the value of *M*_*Z*_*t*. The resultant $M_Z(X,Y) t$ surface map is plotted in Fig. 2c. It is noteworthy that although the magnetic domains of TmIG and TmIG/Pt are well reproduced, the reconstruction slightly overestimates *M*_*Z*_ near the DW due to the left Néel character of the DW.

## Supplementary information

Supplementary Information

## Data Availability

The data that support the findings of this study are available from the corresponding authors on reasonable request.
