# Supplementary material for: High-speed domain wall racetracks in a magnetic insulator
Source: Nat Commun. 2019 Oct 18;10:4750. doi: 10.1038/s41467-019-12676-7 (PMC6802104; doi:10.1038/s41467-019-12676-7)
Supplement: Supplementary file 1 — Supplementary Information [file 41467_2019_12676_MOESM1_ESM.pdf]

Supplementary Information for

High-speed domain wall racetracks in a magnetic insulator

Saül Vélez[1,§,*], Jakob Schaab[1,§], Martin S. Wörnle[1,2], Marvin Müller[1], Elzbieta Gradauskaite[1], Pol Welter[2], Cameron Gutgsell[1], Corneliu Nistor[1], Christian L. Degen[2,*], Morgan Trassin[1,*], Manfred Fiebig[1] and Pietro Gambardella[1,*]

[1] Department of Materials, ETH Zurich, 8093 Zurich, Switzerland

[2] Department of Physics, ETH Zurich, 8093 Zurich, Switzerland

[§] These authors contributed equally

[*]e-mail: saul.velez@mat.ethz.ch (S.V.); degenc@ethz.ch (C.L.D.); morgan.trassin@mat.ethz.ch (M.T.); pietro.gambardella@mat.ethz.ch (P.G.)

Table of contents:

- Supplementary Note 1. Structural and topographic characterization of TmIG and TmIG/Pt films
- Supplementary Note 2. Magnetic characterization of TmIG and TmIG/Pt films
- Supplementary Note 3. Electrical characterization of TmIG/Pt by spin Hall magnetoresistance: determination of the interface quality and the magnetic anisotropy
- Supplementary Note 4. Factors influencing magnetization reversal beyond the Pt current line: DW depinning field, Joule heating and the Oersted field
- Supplementary Note 5. Comparison of magnetization reversal detected by Hall and MOKE measurements in TmIG/Pt
- Supplementary Note 6. Current-driven domain wall motion in TmIG/Pt: supplementary data
- Supplementary Note 7. Scanning NV magnetometry

Supplementary Note 1. Structural and topographic characterization of TmIG and TmIG/Pt films

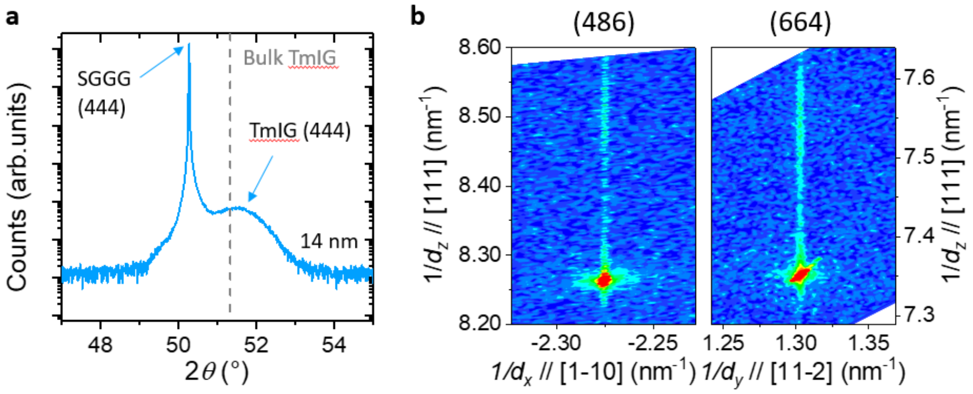

Supplementary Figure 1 | Structural characterization of TmIG thin films. (**a**) Symmetric X-ray diffraction scan around the (444) peak of a SGGG/TmIG(14 nm) sample. The TmIG (444) peak shifts towards higher angles because of a reduction of the out-of-plane lattice constant of the film due to tensile strain (gray dashed line indicates the (444) peak position of bulk TmIG, extracted from Supplementary Ref. 1. (**b**) Reciprocal space maps of the same sample around the (486) and (664) substrate peaks. The in-plane lattice constants of film and substrate along the two perpendicular directions [1-10, 11-2] coincide, confirming full epitaxy. The same strain state is expected for the thinner TmIG(8.3 nm)/Pt sample discussed in the main text as strain is maintained for thicknesses up to ~ 50 nm (see Supplementary Ref. 2). The colour code indicates the intensity of the diffraction peaks, with red (blue) corresponding to maximum (minimum) intensity.

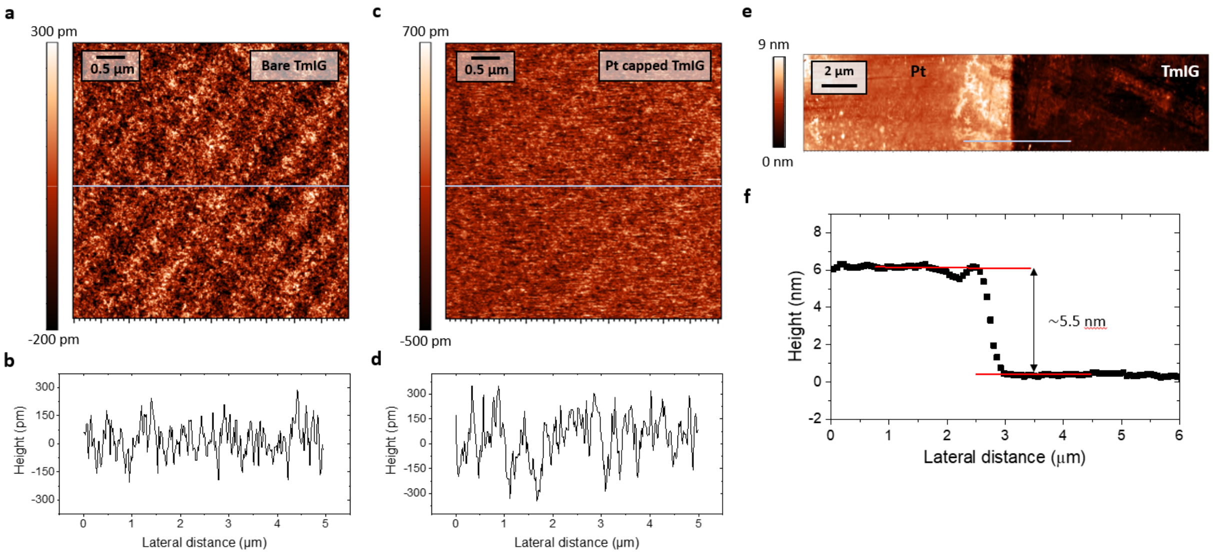

Supplementary Figure 2 | Topographic characterization of TmIG and TmIG/Pt films. (**a**,**c**) Atomic force microscopy (AFM) characterization of the surface topography of a bare TmIG and a Pt-capped TmIG film, respectively. The root mean square (RMS) roughness is on the order of 0.096 and 0.136 nm, respectively. (**b**,**d**) AFM line profiles along the blue lines indicated in panels **a** and **c**, respectively, confirming the low roughness of our TmIG and TmIG/Pt films. (**e**) AFM scan of the sample studied in the main text in a region partially covered with Pt. Some resist residues arising from the lithography process can be seen on both the Pt covered and etched regions. (**f**) Average topography scan of the Pt edge along the blue line indicated in **e**, showing that the step size is on the order of ~5.5 nm. The surface RMS is below 0.2 nm on both the etched and Pt covered regions.

Supplementary Note 2. Magnetic characterization of TmIG and TmIG/Pt films

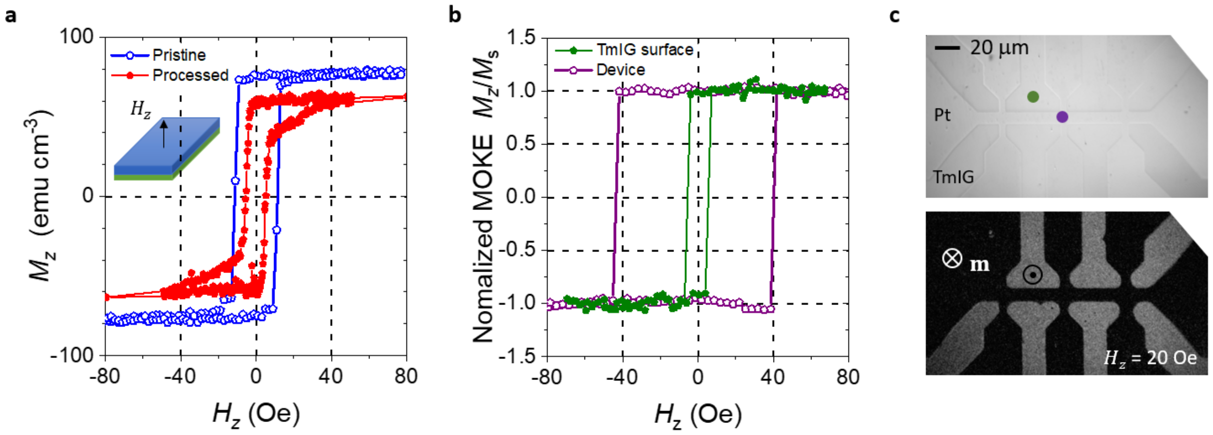

Supplementary Figure 3 | Magnetic characterization of the TmIG(8.3 nm)/Pt(5.0 nm) sample. (**a**) Out-of-plane SQUID measurements performed before (blue) and after (red) patterning of the Pt layer. For calculating M_z, we considered that the effective thickness of the TmIG layer is 7.3 nm after etching. (**b**) Normalized out-of-plane magnetization of TmIG measured by spatially-resolved MOKE microscopy in different regions of the film as indicated by the coloured dots in **c**, top image. (**c**) Top, optical image of a device. Bottom, differential MOKE image taken on the same device at $H_z = 20$ Oe, showing reversed magnetic domains (bright contrast) only around the device. The film was initially fully down magnetized (dark contrast) by applying a magnetic field $H_z = 100$ Oe.

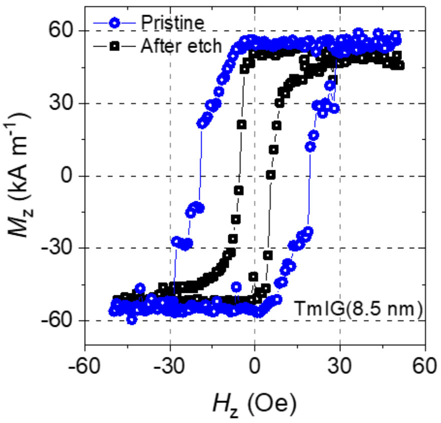

Supplementary Figure 4 | Magnetic characterization of the TmIG(8.5 nm) reference film. Out-of-plane SQUID measurements performed in the film before (blue) and after exposure of the surface to the Ar-plasma (black), which reproduces the exposure of the TmIG surface to the Ar-plasma in the TmIG(8.3 nm)/Pt(5.0 nm) sample during the etching of the Pt layer. For calculating M_z, we considered that the effective thickness of the TmIG layer has been reduced to 7.5 nm after exposure of the surface to the Ar-plasma due to both etching and surface passivation.

Saturation magnetization

The magnetization of TmIG is known to be sensitive to the film thickness as well as to oxygen content. Indeed, a significant reduction of M_{S} compared to bulk values (~ 110 kA m^{-1}, see Supplementary Ref. 3) has been reported to occur for TmIG films below ~ 10 nm thickness[4,5], which can be due to either finite size effects[4] or a slight oxygen deficiency caused by low oxygen pressure during growth[2,6]. The SQUID measurements reported in Supplementary Figs. 3a and 4 show that our TmIG(8.3 nm)/Pt(5.0 nm) and TmIG(8.5 nm) films present a reduced M_{s} compared to the bulk. Importantly, both films exhibit perpendicular magnetic anisotropy, both before and after exposure of the films to Ar-plasma. For the TmIG(8.3 nm)/Pt(5.0 nm) sample, the Ar-plasma was employed to pattern the Pt layer into racetrack devices (see Methods). For the reference TmIG(8.5 nm) film, the surface was exposed to the Ar-plasma in order to reproduce the exposure time of the TmIG surface of the TmIG(8.3 nm)/Pt(5.0 nm) sample during the etching of the Pt layer. The exposure time was set according to the calibrated etching rate of Pt determined using Pt reference films.

For calculating M_{S} from the SQUID measurements, we assume that the effective TmIG thickness of the TmIG/Pt sample is reduced by about 1 nm to 7.3 nm after the etching of the Pt due to partial etching (as evidenced by AFM, see Supplementary Fig. 2) and likely passivation of the top surface of the TmIG layer, as suggested by the comparison of the SQUID data taken for the reference TmIG(8.5 nm) film before and after exposure to the Ar-plasma (see Supplementary Fig. 4), and between the measured M_{S} of the pristine reference TmIG film and the TmIG/Pt sample after etching. By assuming 1 nm reduction of the TmIG thickness, the SQUID measurements indicate that the saturation magnetization is $M_{\mathrm{S}} = (55 \pm 2)$ kA m^{-1} and $M_{\mathrm{S}} = (52 \pm 3)$ kA m^{-1} in the reference TmIG film before and after the Ar-plasma exposure (Supplementary Fig. 4), respectively, and that $M_{\mathrm{S}} = (59 \pm 2)$ kA m^{-1} in the TmIG/Pt sample after etching (red curve, Supplementary Fig. 3a). Note that some portion of the film surface is still covered with Pt after etching, which explains the slightly larger M_{S} measured for the Pt/YIG sample (see also the NV magnetometry data below).

The saturation magnetization in TmIG/Pt is larger than in TmIG (see Supplementary Fig. 3a). From SQUID measurements, we find that in TmIG/Pt the saturation magnetization is $M_{\mathrm{S}} = (77 \pm 2)$ kA m^{-1}, which corresponds to a difference in M_{S} of ~ 20 kA m^{-1} with respect to TmIG. This difference is likely to be due to a partial polarization of the Pt atoms in proximity with TmIG. By assuming that the first three atomic layers of Pt in contact with TmIG get polarized, we estimate that this difference is equivalent to a magnetic moment per Pt atom of about 0.4 μ_{B}.

The surface magnetization $M_z t$ obtained by NV magnetometry measurements compare well with the saturation magnetization measured by SQUID. By dividing the measured $M_z t$ of the TmIG/Pt sample after etching (see Fig. 2) by $t = 8.3$ nm and $t = 7.3$ nm for the TmIG/Pt and TmIG regions, respectively, we obtain $M_{\mathrm{S}} = (75 \pm 2)$ kA m^{-1} and $M_{\mathrm{S}} = (50 \pm 3)$ kA m^{-1}. Regarding the reference sample, the surface magnetization measured before the exposure of the surface to the Ar-plasma was $M_z t = (48.9 \pm 3.2)$ μ_{B} nm^{-2}, which corresponds to $M_{\mathrm{S}} = (53 \pm 4)$ kA m^{-1} (see also Supplementary Note 7).

Coercive field

The coercive field measured by SQUID for the TmIG(8.3 nm/Pt(5.0 nm) sample changes from H_c $\sim$ 13 to $\sim$ 6 Oe after etching and patterning of the Pt film (see Supplementary Fig. 3a). A reduction of H_c from ~ 19 to ~ 6 Oe, is also observed in the reference TmIG(8.5 nm) layer after exposure to the Ar-plasma (see Supplementary Fig. 4). Although the coercive field is reduced due to the etching process, the saturation field of TmIG(8.5 nm) is similar to the one measured before etching. However, the saturation field of TmIG/Pt increases substantially after patterning the Pt layer into Hall bar-like racetrack devices, which indicates that having a combination of etched TmIG and unetched TmIG/Pt regions at the surface of the film influences the magnetization reversal process.

Spatially-resolved wide-field MOKE measurements performed on the TmIG/Pt sample after patterning of the Pt layer show that the coercive field in the region underneath the Pt devices is usually much larger than in the area next to them. Supplementary Figures 3b,c show representative MOKE measurements taken on an area partially covered by Pt during field-driven magnetization reversal. Starting from a fully magnetized state with $M_z < 0$, we observe that most of the area surrounding the device has already switched at $H_z = +20$ Oe (white contrast in Supplementary Fig. 3c, bottom image), whereas the area covered by Pt remains magnetized down (dark contrast). Further increase of the magnetic field results in a sharp switching of the whole area underneath the Pt-device at $H_z \sim +40$ Oe (see violet curve in Supplementary Fig. 3b; note that the coercive field is similar to the one obtained by AHE-like measurements, see Fig. 1b). These measurements suggest that, besides changes of the magnetic anisotropy that may occur between the TmIG/Pt and TmIG regions, the edges of the Pt pattern act as pinning centres that prevent the free expansion of domains and domain walls during a magnetic field sweep, thus influencing the magnetization reversal dynamics. Note that different coercive fields were measured in different regions and devices across the film, which we attribute to the particular local distribution of defects.

These observations support the idea that the low-concentration of pinning centres present in TmIG is the most relevant ingredient for the square-like magnetization reversal observed in the pristine TmIG/Pt (blue dots in Supplementary Fig. 3a and Supplementary Fig. 4). The more progressive reversal of the magnetization measured on the same sample after etching of the Pt layer (red dots in Supplementary Fig. 3a), which starts at a lower applied magnetic field, is consistent with the presence of additional surface defects introduced by the etching process, which act as both domain nucleation and domain wall pinning centres, resulting in a more complex magnetization reversal process. The observed reduction in the coercive field of the TmIG reference film after Ar-plasma exposure, and the progressive approach to saturation upon magnetization reversal, with similar saturation field than the one measured before etching, is consistent with the interpretation that nucleation centres are likely introduced along the surface, which modify the magnetization reversal process, but without resulting in a noticeable modification of the magnetic anisotropy or saturation magnetization of the film.

Supplementary Note 3. Electrical characterization of TmIG/Pt by spin Hall magnetoresistance: determination of the interface quality and the magnetic anisotropy

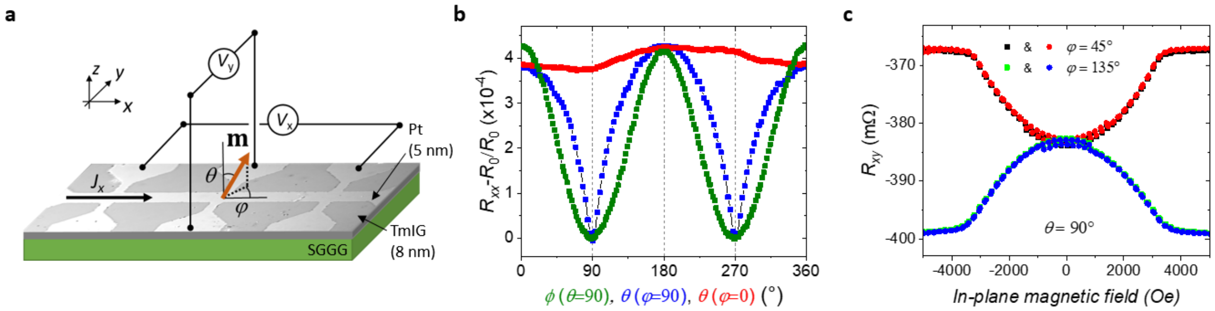

Supplementary Figure 5 | Electrical characterization of the spin Hall magnetoresistance and magnetic anisotropy of TmIG/Pt. (**a**) Optical image of a patterned Pt Hall bar on the TmIG(8.3 nm) film presented in the manuscript with superposed electric wiring, coordinate system and the definition of the polar angles θ, φ, and the magnetization vector $\mathbf{m}$. (**b**) Magnetoresistance measurements performed for $\mathbf{m}$ rotating in the xy- (green, $\theta = 90°$ and variable φ), yz- (blue, fixed $\varphi = 90°$ and variable θ), and xz-plane (red, $\varphi = 0°$ and variable θ). The direction of $\mathbf{m}$ is controlled by rotating the sample in a magnetic field of amplitude 5 kOe. The angular dependences display the symmetry expected for spin Hall magnetoresistance[7]. (**c**) Hall resistance R_{xy} measurements performed by sweeping the magnetic field in the plane of the sample ($\theta = 90°$) at $\varphi = 45°$ (black and red dots, trace and retrace) and $\varphi = 135°$ (green and blue dots, trace and retrace). The variation of R_{xy} with the applied magnetic field $\mathbf{H}$ is due to the canting of $\mathbf{m}$ –from the out-of-plane easy axis– towards $\mathbf{H}$. The saturation of R_{xy} at large magnetic fields indicates that $\mathbf{m}$ is saturated in the plane of the film, thus giving an estimation for the magnetic anisotropy field of the TmIG film of about $H_\mathrm{k} \sim 3$ kOe.

According to the theory of the spin Hall magnetoresistance (SMR), the first harmonic response of the magnetoresistance in TmIG/Pt can be described as[7,8]:

$$R_{xx} = \frac{V_x}{I_x} = R_0 + \Delta R^{\mathrm{SMR}}(1 - \sin^2\theta \sin^2\varphi), \tag{1}$$

$$R_{xy} = \frac{V_y}{I_x} = \frac{W}{2L}\frac{\Delta R^{\mathrm{SMR}}}{2}\sin^2\theta \sin 2\varphi + R_\mathrm{H}^{\mathrm{AHE,SMR}}\cos\theta + R_\mathrm{H}^{\mathrm{OHE}}H_z, \tag{2}$$

where θ and φ denote the polar and azimuthal angle of the normalized magnetization vector $\mathbf{m} = \mathbf{M}(x, y, z)/M_\mathrm{s}$ collinear to the applied field $\mathbf{H}$ (see Supplementary Fig. 5a), $W/2L = 0.1$ is the width/length ratio of the measuring configuration of the Hall bar (i.e., 10/100, see Supplementary Fig. 5a), R_0 is the longitudinal base resistance, ΔR^{SMR} the SMR amplitude, $R_\mathrm{H}^{\mathrm{AHE,SMR}}$ the anomalous Hall-like SMR amplitude, and $R_\mathrm{H}^{\mathrm{OHE}}H_z$ the ordinary Hall resistance. Supplementary Fig. 5b shows the normalized $(R_{xx} - R_0)/R_0$ angular dependent magnetoresistance measurements performed in our sample with $\mathbf{m}$ rotating along the three main planes of the sample. R_0 is determined to be $\sim 640\ \Omega$. The change in resistance follows the expected behaviour for SMR[7,9]: i) a $\sin^2\theta$ dependence for $\mathbf{m}$ rotating in the plane of the film (green, $\theta = 90°$), ii) a distorted $\sin^2\theta$ dependence for $\mathbf{m}$ rotating in

the yz-plane (blue, $\varphi = 90°$) due to the strong demagnetizing field, and iii) no significant change when $\mathbf{m}$ rotates in the xz-plane (red, $\varphi = 0°$). The SMR amplitude $\Delta R^{\mathrm{SMR}}/R_0 \approx 4.1 \times 10^{-4}$ agrees with previously reported values in TmIG/Pt structures[8,10–12]. From the Hall measurements shown in Supplementary Fig. 5c, as well as Fig. 1b of the main text, we estimate the $R_{\mathrm{H}}^{\mathrm{AHE,SMR}}$ amplitude to be ~ -0.47 mΩ and the ratio $\frac{R_{\mathrm{H}}^{\mathrm{AHE,SMR}}}{R_0}\frac{2L}{W} \sim -0.73 \times 10^{-5}$, which is also consistent with earlier reports in TmIG/Pt structures[4,8,10–12]. From these measurements, we can estimate the real and imaginary parts of the spin-mixing conductance $G^{\uparrow\downarrow} = G_{\mathrm{r}} + iG_{\mathrm{i}}$ using the following expressions[7,9]:

$$\frac{\Delta R_{\mathrm{SMR}}}{R_0} = \theta_{\mathrm{SH}}^2 \frac{\lambda_{\mathrm{N}}}{d_{\mathrm{N}}} \ \mathrm{Re}\left[\frac{2\lambda_{\mathrm{N}}\ G^{\uparrow\downarrow}\ \tanh^2\frac{d_{\mathrm{N}}}{2\lambda_{\mathrm{N}}}}{\sigma_{\mathrm{N}}+2\lambda_{\mathrm{N}}\ G^{\uparrow\downarrow}\coth\frac{d_{\mathrm{N}}}{\lambda_{\mathrm{N}}}}\right], \tag{3}$$

$$\frac{R_H^{\mathrm{AHE,SMR}}}{R_0}\frac{2L}{W} = -\theta_{\mathrm{SH}}^2 \frac{\lambda_{\mathrm{N}}}{d_{\mathrm{N}}} \ \mathrm{Im}\left[\frac{2\lambda_{\mathrm{N}}\ G^{\uparrow\downarrow}\tanh^2\frac{d_{\mathrm{N}}}{2\lambda_{\mathrm{N}}}}{\sigma_{\mathrm{N}}+2\lambda_{\mathrm{N}}\ G^{\uparrow\downarrow}\coth\frac{d_{\mathrm{N}}}{\lambda_{\mathrm{N}}}}\right], \tag{4}$$

where θ_{SH} is the spin-Hall angle, λ_N the spin diffusion length, $d_{\mathrm{N}} = 5.0$ nm the thickness and $\sigma_{\mathrm{N}} = 3.13 \times 10^6$ Ω m the conductivity of the Pt layer. From the conductivity we estimate that λ_{N} ~2.0 nm and $\theta_{\mathrm{SH}} \sim 0.049$ (Supplementary Ref. 13). By considering that $G_{\mathrm{r}} \gg G_{\mathrm{i}}$ (Supplementary Ref. 14), Supplementary Eqs. (3) and (4) yield $G_{\mathrm{r}} \sim 1.1 \times 10^{15}$ Ω^{-1} m^{-2} and $G_{\mathrm{i}} \sim 3.4 \times 10^{13}$ Ω^{-1} m^{-2}, with a corresponding ratio $G_{\mathrm{r}}/G_{\mathrm{i}} \sim 32$, which is consistent with literature values for magnetic insulator/heavy metal bilayers[8,11,12,15,16] and confirms the high quality of the TmIG/Pt interface.

Supplementary Note 4. Factors influencing magnetization reversal beyond the Pt current line: DW depinning field, Joule heating and the Oersted field

As presented in the manuscript, a precise control of the magnetic moment underneath the Pt current line can be achieved in a continuous TmIG film by applying current pulses in the Pt line without altering the magnetic moments of the surrounding region (Fig. 1d). However, the extremely small depinning field of the DWs in TmIG ($\sim$ 1-2 Oe, see Supplementary Fig. 6) makes the current-induced dynamics extremely sensitive to the presence of out-of-plane magnetic fields. Supplementary Fig. 7 shows a sequence of differential MOKE images taken during forward switching in the presence of an out-of-plane field $H_z \sim 2$ Oe, showing that, in contrast to what is observed in Fig. 1d and Fig. 3b, the switched domain clearly extends away from the Pt current line.

The generation of out-of-plane Oersted fields at large current densities ($J_x \sim 10^8$ A cm^{-2} or larger) also favors that the switched domain slightly extends away from the edges of the Pt current line (for about a couple of μm). This is seen in Supplementary Fig. 7, image #7, where the switched domain extends more on the bottom side at the edge of the Pt current line, where the Oersted field favors the forward switching. The Oersted field has an even stronger influence on the switching dynamics during backward switching, as seen in Supplementary Fig. 8.

Current pulses at large current densities also result in a substantial Joule dissipation, which influences the dynamics of the switching process. For instance, Joule heating can lead to the nucleation of multiple domains during a forward switching event (Supplementary Fig. 9) instead of domain nucleation at a defect site followed by current-driven DW motion (Fig. 3b), which occurs at moderate current densities. For long pulses ($t_\mathrm{p} \sim 1$ μs or larger), Joule heating can also favor that the switched domain extends away from the Pt current line.

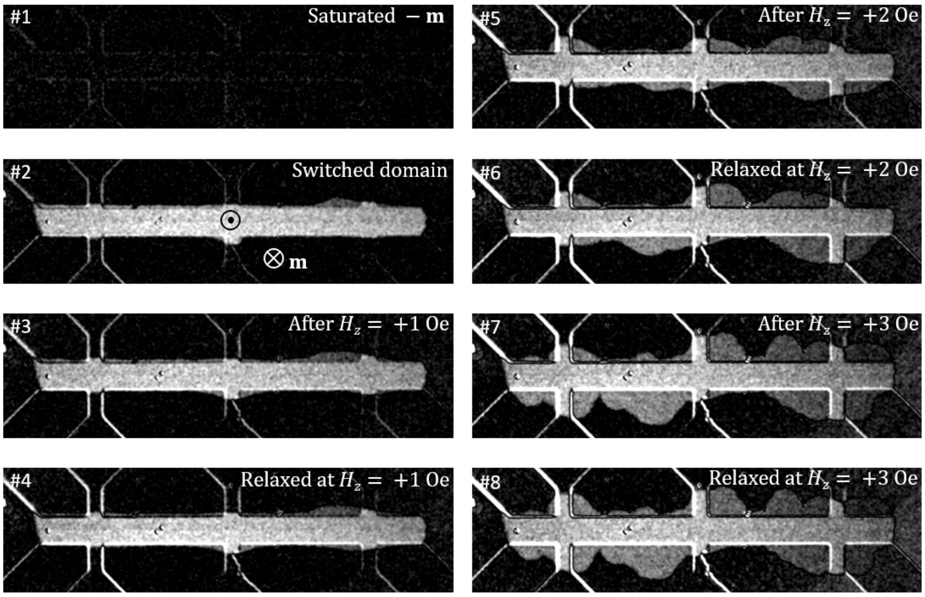

Supplementary Figure 6 | Evaluation of the domain wall depinning field. Sequence of differential MOKE images. #1, The TmIG film is initially satured $-\mathbf{m}$ by applying $H_z = -100$ Oe (dark contrast). #2, The magnetization underneath the Pt current line is switched to $+\mathbf{m}$ (bright contrast) by applying a forward switching current pulse. #3 to #8, An out-of-plane field $+H_z$ is applied and the evolution of the DWs of the $+\mathbf{m}$ domain tracked with time and strength of the magnetic field. Images #3/5/7 are taken right after applying $H_z = +1/2/3$ Oe, whereas images #4/6/8 are the subsequent MOKE images taken after waiting 20 sec at the same field. A first tiny DW displacement is already observed for $H_z = +1$ Oe. At $H_z = +2$ Oe the DW is clearly unpinned and the domain extends significantly through the TmIG film. We thus determine that the DW depinning field in TmIG is on the order of $\sim$1-2 Oe.

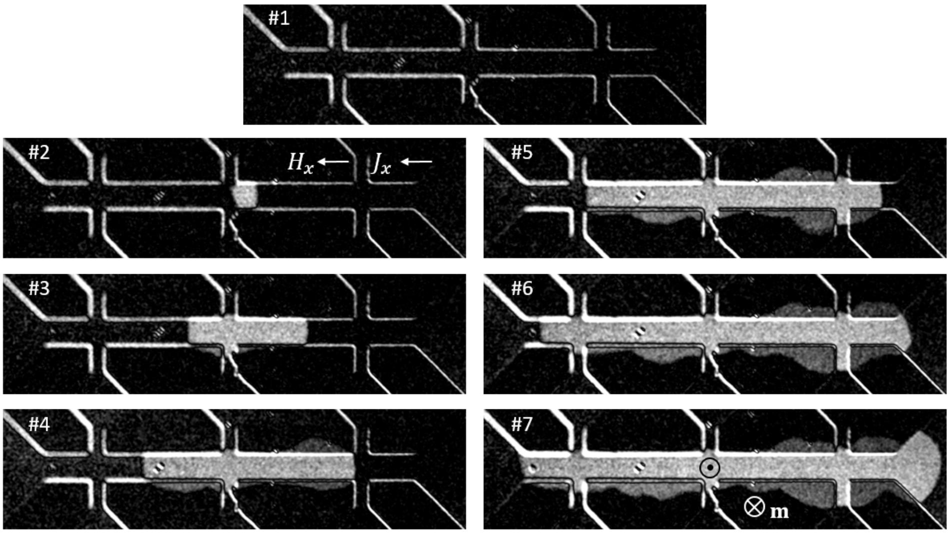

Supplementary Figure 7 | Forward switching in the presence of an out-of-plane field $\boldsymbol{H_z}$. Sequence of differential MOKE images taken during a series of forward switching current pulses of $J_x = 0.9 \times 10^8$ A cm^{-2} and $t_\mathrm{p} = 75$ ns in the presence of an external field of 400 Oe applied along the Pt current line at an angle of $\sim 0.3°$ with respect to the plane of the film, which results in an out-of-plane field $H_z \sim 2$ Oe. The film is initially saturated down ($-\mathbf{m}$, dark contrast). Bright contrast indicates the TmIG region that has switched to $+\mathbf{m}$. The finite H_z favors the spreading of the switched domain away from the current line. These measurements indicate that the accuracy in the alignment of the sample for the measurements presented in the manuscript is better than $0.1°$.

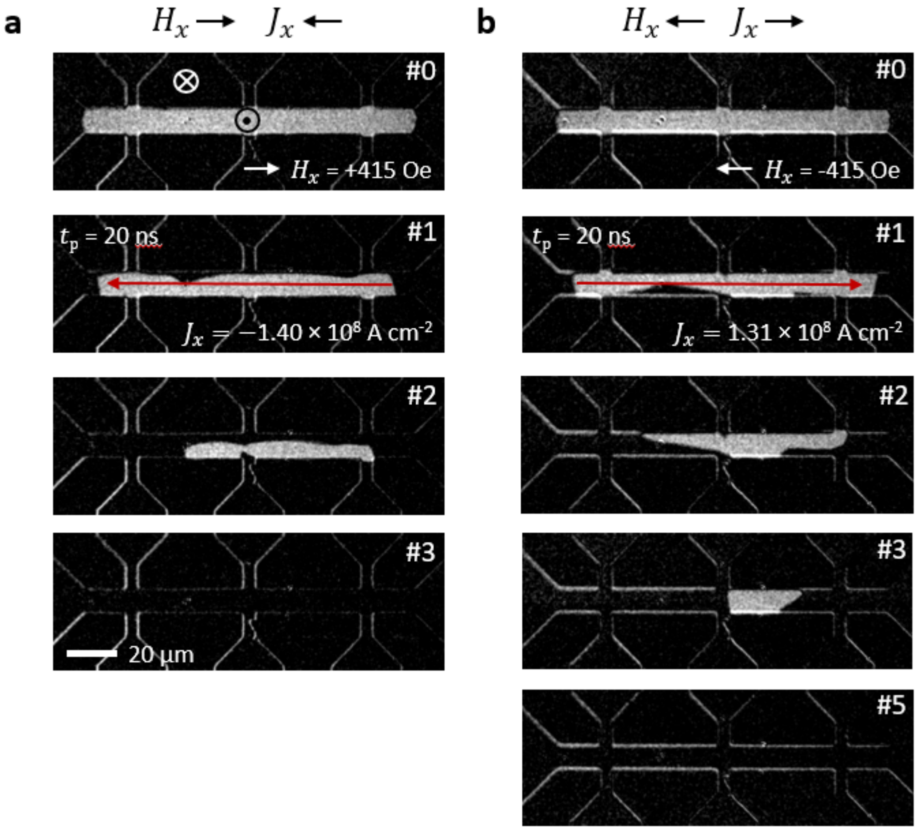

Supplementary Figure 8 | Switching dynamics at large current densities: influence of the Oersted field. (**a**,**b**) Different examples of the dynamics of the DWs upon the application of a series of backward switching current pulses at large current densities ($> J_x = 1.0 \times 10^8$ A cm^{-2}). The current pulses produce out-of-plane Oersted fields of opposite polarity at the opposite edges of the Pt current line (i.e., bottom and top edges in the images). For a negative current, a positive (negative) out-of-plane Oersted field is generated at the bottom (top) edge of the Pt current line, and vice versa upon inverting the current direction. These Oersted fields can influence the switching dynamics given the extremely small DW depinning field in TmIG ~1-2 Oe (see Supplementary Fig. 6). Therefore, for current pulses inducing the contraction of a $+\mathbf{m}$ domain (bright regions) in a $-\mathbf{m}$ medium (black regions), the negative out-of-plane Oersted field generated at the top (bottom) edge of the Pt current line for a negative (positive) current is sufficient to induce the depinning of the DW at the edge, pushing it towards the centre of the line. As a result, the domain contraction has both transverse and longitudinal components, driven by the SOT and Oersted field, respectively (see images in **a** and **b**).

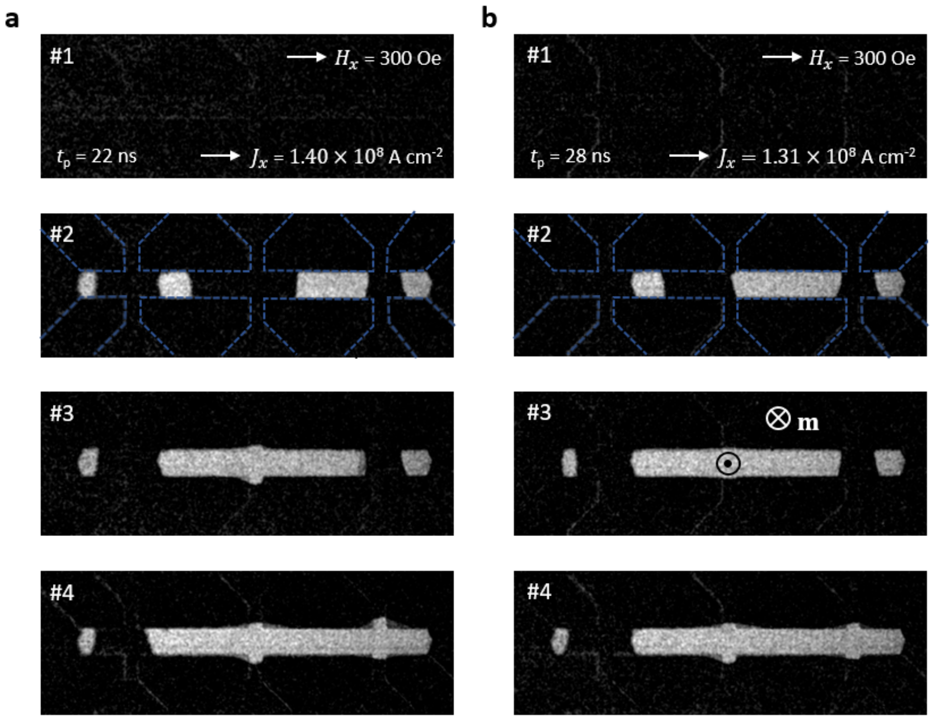

Supplementary Figure 9 | Multiple domain nucleation at large current densities. (**a**,**b**) Examples of multiple nucleation of domains at large current densities. Images #1 to #4 correspond to a series of forward switching current pulses. In contrast to low current densities, where nucleation takes place at a defect site (see Fig. 3b and Supplementary Fig. 6), here the nucleation takes place preferentially in the region comprised between two adjacent Hall crosses (not right at the cross, see #2), where the current density is larger. Bright and dark contrast correspond to regions of **+m** and **-m**, respectively.

Supplementary Note 5. Comparison of magnetization reversal detected by Hall and MOKE measurements in TmIG/Pt

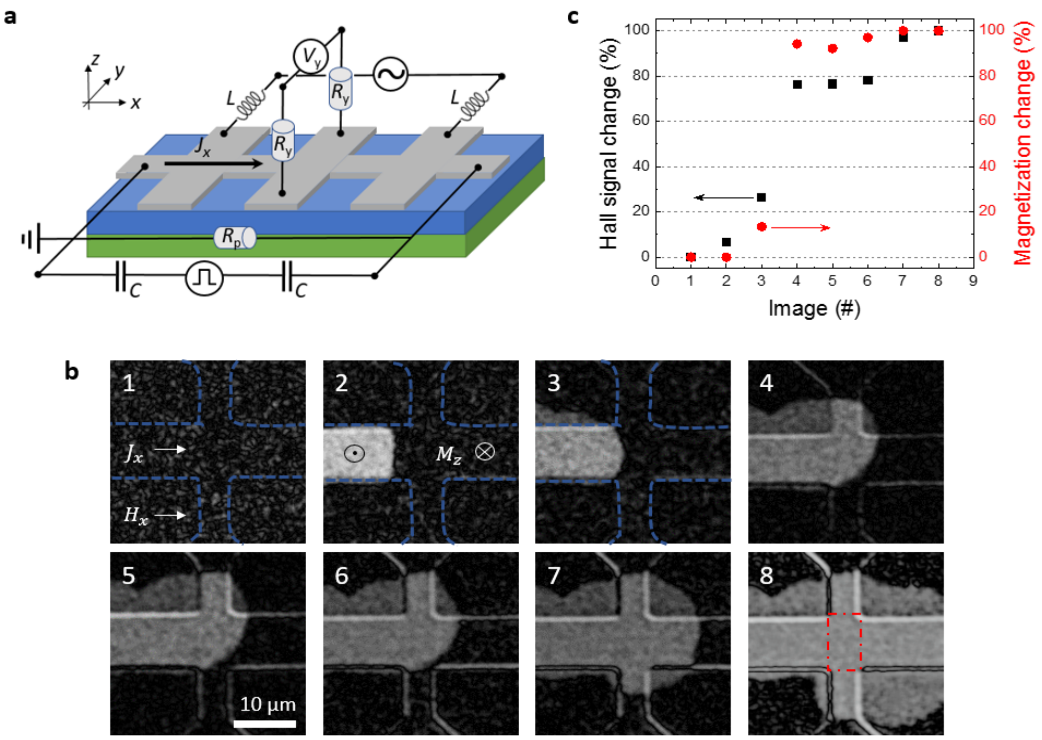

Supplementary Figure 10 | Simultaneous Hall and MOKE detection of magnetization reversal in TmIG/Pt. (**a**) Measurement scheme. Current pulses are applied via two capacitors (C = 150 nF) to the Hall bar, which allows current pulses faster than ~ 1 µs to completely pass through while filtering low-frequency signals. The current line is shunted by a 50 Ohm resistance R_p for impedance matching. The AC current source for the harmonic Hall measurements (f = 11 Hz) is connected via inductors ($L = 440$ µH), which filter signals faster than ~ 1 µs. The voltage probes for V_y are connected through two resistors $R_y = 100$ kΩ, which prevent damage of the Hall contacts from the current pulses. (**b**) Tracking of the current-induced expansion of a $+\mathbf{m}$ domain (bright area) in a down magnetized medium ($-\mathbf{m}$, black). Between each image, a current pulse of $J_x = 7.5 \times 10^7$ A cm^{-2} and adjustable pulse length (50 to 500 ns) is applied to slowly drive the DW through the Hall cross located at the centre of the Hall bar (see **a**). The magnetic field is set at $H_x = 100$ Oe. (**c**) Simultaneous measurement of the relative change in the Hall signal (black) together with the measurement of the magnetization change by direct MOKE imaging (red) for the sequence of images shown in **b**. The change in magnetization corresponds to the red dashed area indicated in image #8. The change in the Hall resistance is due to $R_\mathrm{H}^\mathrm{AHE,SMR}$ (see Supplementary Note 3 and Supplementary Fig. 5), which reflects the change of m_z. Comparison of the Hall and MOKE data show that the electrical reading of the magnetic state of TmIG is only sensitive to changes in the vicinity of the Hall cross. However, note that spreading of domains beyond the Hall cross area also influence $R_\mathrm{H}^\mathrm{AHE,SMR}$. While wide-field MOKE measurements allow spatially-resolved detection of the magnetic state of TmIG in any area of the film (only limited by optical resolution), electrical measurements do not allow to determine the DW position and displacement with precision after the application of current pulses.

Supplementary Note 6. Current-driven domain wall motion in TmIG/Pt: supplementary data

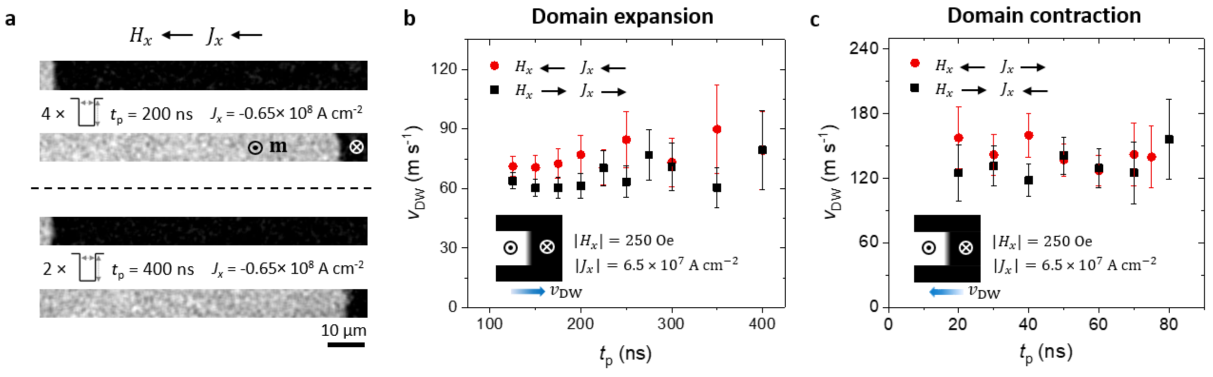

Supplementary Figure 11 | Dependence of v_{DW} on the pulse length. (**a**) Differential MOKE images showing the displacement of an up-down DW after the application of $N = 4$ current pulses of $t_{\mathrm{p}} = 200$ ns and $N = 2$ pulses of $t_{\mathrm{p}} = 400$ ns. $H_x = -250$ Oe and $J_x = -6.5 \times 10^7$ A cm^{-2} in both cases. The same displacement Δx is observed for both sequences of current pulses, leading to the same estimate for the DW speed $v_{\mathrm{DW}} = \Delta x \,/\, (N_{\mathrm{p}}\, t_{\mathrm{p}})$ (see Methods). (**b**) Systematic evaluation of v_{DW} for an up-down DW during domain expansion as a function of t_{p} in the regime where DWs move upon the application of current pulses but are not strongly influenced by Joule heating. (**c**) Same as **b** for domain contraction. Within the experimental error, v_{DW} remains roughly constant although t_{p} changes by a factor of ~ 4, which allows us to conclude that the DW speed is accurately evaluated upon changing t_{p} and not influenced by DW inertia. We attribute the slight increase of v_{DW} at large t_{p} to Joule heating. For the measurements presented in Fig. 4 we reduced the influence of Joule heating by measuring v_{DW} at the lower edge of the parameter space t_{p} at any given H_x, J_x combination. Joule heating (as well as the Oersted field, see Supplementary Note 4) is found to influence the DW dynamics and magnetization switching at current densities $\gtrsim 1 \times 10^8$ A cm^{-2}. The error bars in **b** and **c** indicate the uncertainty in estimating v_{DW} from the DW displacement obtained for a sequence of current pulses.

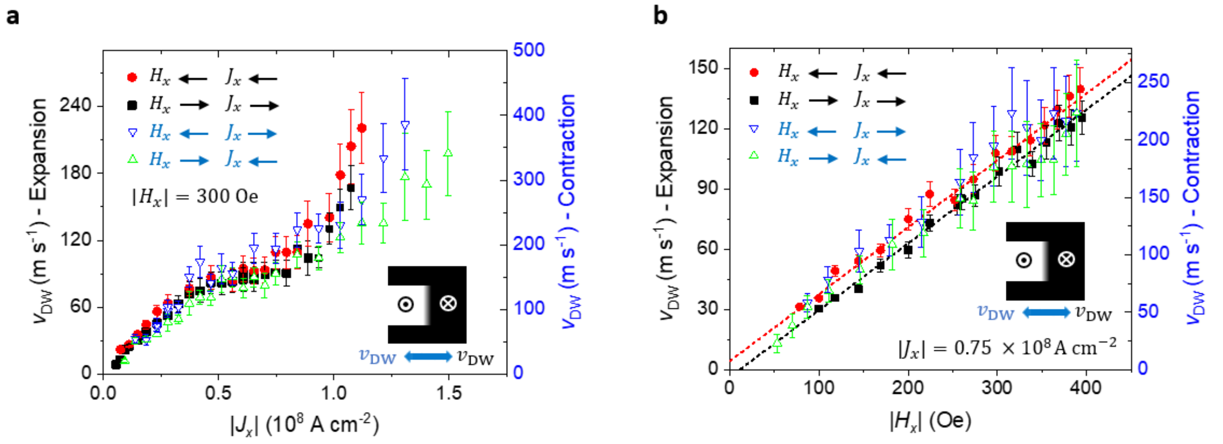

Supplementary Figure 12 | Scaling of v_{DW} for expanding and contracting domains. (**a**) Same data as in Figs. 4a and 4b, i.e., DW velocity as a function of $|J_x|$ for domain expansion (full black square and red circle dots; left vertical axis) and domain contraction (open green up and blue down triangles; right vertical axis), plotted together. (**b**) Same data shown in Figs. 4c and 4d plotted together. The same labelling as in **a** is employed. Note that in both **a** and **b,** the v_{DW}-Contraction axis (blue) is 1.75 times larger than the v_{DW}-Expansion axis (black). The v_{DW} data for expansion and contraction scale on top of each other in the flow and saturation regimes, i.e., for the entire range of H_x in **b** and for $|J_x| \lesssim 0.9 \times 10^8$ A cm^{-2} in **a**. This scaling shows that the DW mobility is a factor 1.75 larger for the contraction case relative to the expansion case, independently of field and current. As discussed in the main text, as well as in the Supplementary Note 4 and Supplementary Fig. 11, Joule heating influences the DW dynamics, resulting in an upturn of the DW velocity. The steeper increase of $v_{\mathrm{DW}}(J_x)$ observed for domain expansion relative to domain contraction in this high current density regime is attributed to the larger dissipated heat in the former case, because longer current pulses t_{p} are employed for moving DWs upon expansion (see Fig. 3d). The error bars account for the uncertainty in estimating v_{DW} from each measurement.

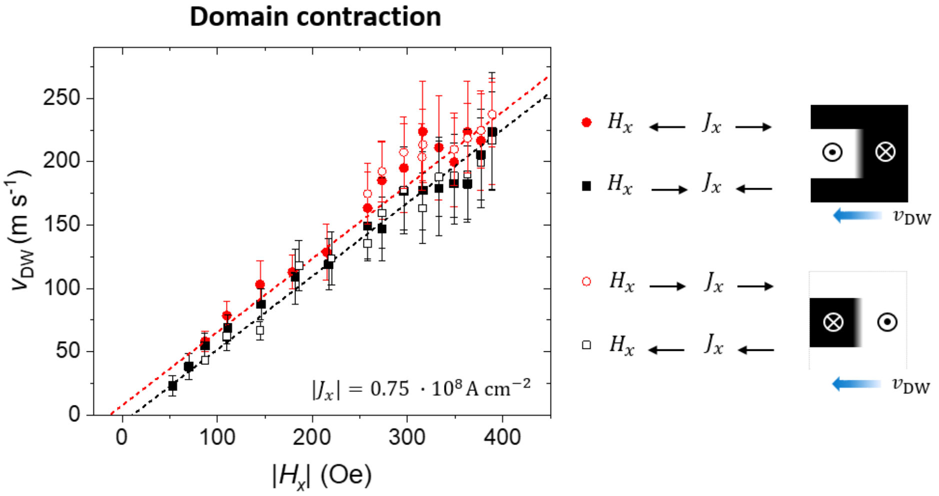

Supplementary Figure 13 | Comparison of v_{DW} between an up-down and a down-up domain wall. v_{DW} as a function of H_x for a down-up DW, and for the two combinations of H_x and J_x that lead to a domain contraction (open symbols), together with the data presented in Fig. 4d for a contracting up-down DW (solid symbols). Within the experimental error, both DWs exhibit a similar larger/slower speed when moving against/along J_x, confirming the weak negative DMI found in TmIG/Pt. See also Supplementary Fig. 14, where the DW structure and current-induced DW motion is discussed for both up-down and down-up DWs and for all four combinations of H_x, J_x directions. The error bars account for the uncertainty in estimating v_{DW} from each measurement.

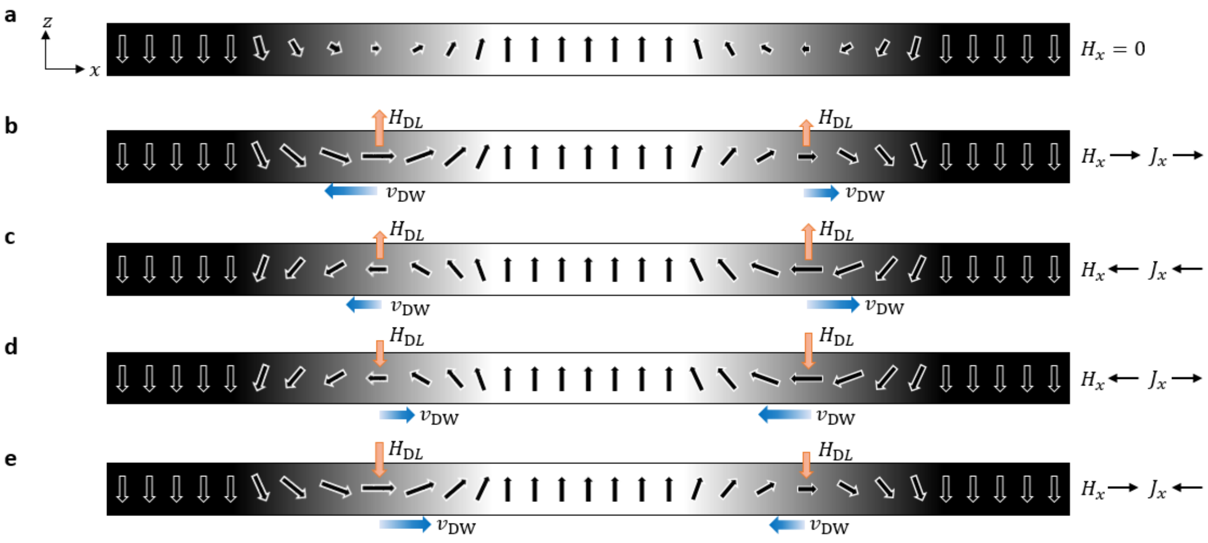

Supplementary Figure 14 | Schematics of the domain wall structure and current-induced domain wall motion in the presence of H_x and DMI. (**a**) Cross-sectional schematics (xz-plane) of a down-up and an up-down DW (positioned along the y-direction) in TmIG/Pt illustrating its internal structure in the absence of H_x. The finite left-handed Néel chirality of the DWs reflects the negative DMI found in TmIG/Pt (see Fig. 2 and Fig. 4c,d). (**b**-**e**) The internal magnetization of the DWs can be controlled by applying a magnetic field H_x exceeding the effective DMI field H_{DMI}, where the latter is positive (negative) for a down-up (up-down) DW (see **a**). Consequently, upon the application of H_x, the internal magnetization of up-down and down-up DWs has either a larger or smaller projection on the direction of J_x. As the amplitude of the damping-like SOT is maximal when the DW magnetization is parallel or antiparallel to J_x, up-down and down-up DWs move at different speed. The damping-like SOT is represented by the corresponding effective field H_{DL} in the figure. Application of current pulses drive the DWs as indicated for all four possible combinations of H_x and J_x applied (see **b**-**e**). DWs are driven at larger (smaller) velocities when moving against (towards) J_x, which is consistent with the results presented in Fig. 4 and Supplementary Fig. 13.

Supplementary Note 7. Scanning NV magnetometry

Definition of the polar angles $\boldsymbol{\theta}_{\mathbf{NV}}$ and $\boldsymbol{\phi}_{\mathbf{NV}}$

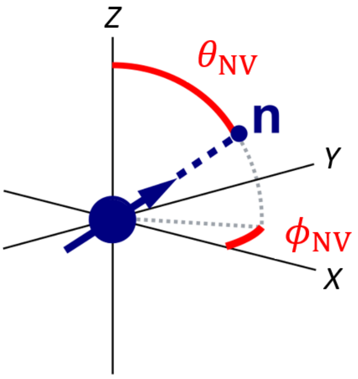

Supplementary Figure 15 | Definition of the polar angles $\boldsymbol{\theta}_{\mathbf{NV}}$ and $\boldsymbol{\phi}_{\mathbf{NV}}$ with respect to the laboratory coordinate frame (see Fig. 2). $\theta_{\rm NV} = (55 \pm 2°)$ is determined by sample fabrication. Analysis of the stray field $B_{\rm NV}$ allows us to determine that $\phi_{\rm NV} = (83 \pm 3)°$.

Determination of the stand-off distance $\boldsymbol{d}$:

The sample-to-sensor distance d is inferred *in situ* from the change in the stray field $B_{\rm NV}$ at the TIG/Pt edge (see Fig. 2b) due to the different magnetic response of TmIG relative to TmIG/Pt (see Fig. 2c), which we mainly attribute to a proximity induced polarization of the Pt layer (see second dataset taken in a reference TmIG film). The change in magnetization gives rise to a stray field that is added to the field produced by the DW. In order to disentangle both fields we use a differential approach by shifting the images along the y-axis and subtracting them from each other, see Supplementary Fig. 16. In this way we cancel the domain wall stray field and end up with a stray field showing only two Pt edges. The magnetic stray field emanating from the Pt edges, with the image orientated such that they are aligned with the y-direction, can be described analytically as

$$B_x = \frac{\mu_0 M_{\rm s,Pt} t}{2\pi}\left[\frac{d - x_1}{d^2 + (x - x_1)^2} - \frac{d - x_0}{d^2 + (x - x_0)^2}\right]$$

$$B_y = 0$$

$$B_z = -\frac{\mu_0 M_{\rm s,Pt} \cdot t}{2\pi}\left[\frac{x - x_1}{d^2 + (x - x_1)^2} - \frac{x - x_0}{d^2 + (x - x_0)^2}\right], \qquad (5)$$

where x_0, x_1 denote the edge positions and $M_{\rm s,Pt} t$ is the surface magnetization of Pt. By fitting this model to the experimental line scan we can extract a value for the stand-off distance d and $M_{\rm s,Pt}$. Further fit parameters are the edge positions and the azimuth angle of the NV vector orientation. To improve statistics and estimate the error, we fit several line scans across the stripe using the same starting parameters and extract the mean value and standard error. For the image shown in Fig. 2b and Supplementary Fig. 16, we obtain $d = 104 \pm 3.3$ nm. Further stand-off distance measurements on a separate calibration sample (Pt/Co/AlO_x) before and after the domain wall measurement are in

good agreement with this value. To compensate for the fact that we have not included the height profile of the stripe as well as the Dzyaloshinskii-Moriya interaction, we deliberately over-estimated the error in the stand-off distance to be larger than the fit errors and set it to 5 nm.

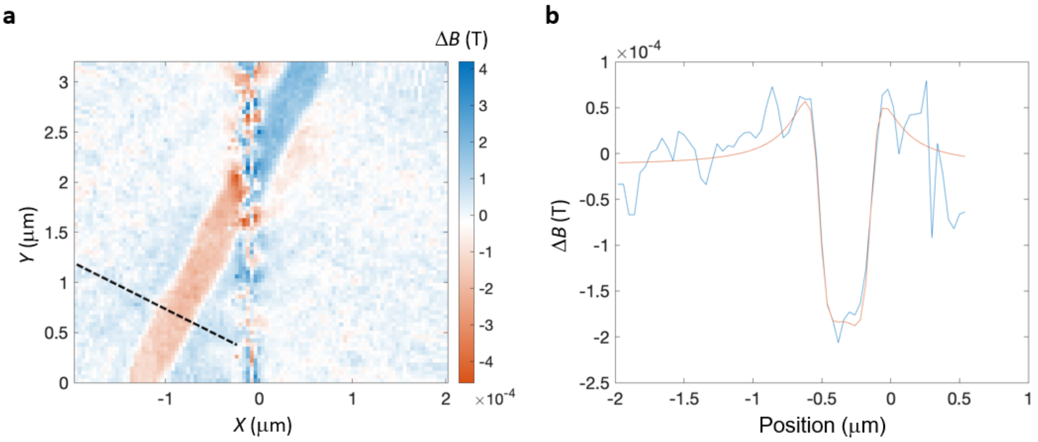

Supplementary Figure 16 | In situ calibration of the NV stand-off distance on TmIG/Pt. (**a**) Differential stray field image. The plotted image was obtained by subtracting two original images (Fig. 2b) that were shifted relative to each other by 800 nm along the Pt edge. (**b**) Representative line cut of the differential image along the dashed line indicated in **a**, together with the fit according to Supplementary Eq. (5).

Fits of the domain wall magnetization profile:

We fit several line scans of the DW in TmIG and TmIG/Pt using the same starting fit parameters as described in Methods. The resulting fit parameters p_i are averaged, with weights according to their variance. The sum is taken over all line scans corresponding to the selected region. The nominal value of the parameters are calculated as $\bar{p}_\mathrm{i} = \sum_{\mathrm{k}=1}^{\mathrm{N}} w \cdot p_{\mathrm{i,k}}$, where *k* indicates the number of the scan. The standard deviation of each parameter is then $\sigma_\mathrm{i} = \sqrt{\sum_{\mathrm{k}=1}^{\mathrm{N}} \left(w \cdot p_{\mathrm{i,k}}^2 - \bar{p}_{\mathrm{i,k}}^2\right) + \left(\delta d \frac{\partial \bar{p}_\mathrm{i}}{\partial \bar{d}}\right)^2}$. The last term describes the uncertainty due to the error of the stand-off distance δd. Since the fitting algorithm assumes the stand-off distance to be fixed, it is not included in the fitting results directly. We estimate $\frac{\partial \bar{p}_\mathrm{i}}{\partial \bar{d}}$ via finite differences by repeating the fitting procedure with the stand-off distance equal to the error boundaries $d^\pm = \bar{d} \pm \delta d$ with $\bar{d} = 104$ nm.

Second dataset: reference TmIG film

We recorded a second dataset with a different scanning probe ($d = 66 \pm 6$ nm) on an unprocessed TmIG film of thickness 8.5 nm. Analysis of this second dataset yielded a chiral angle of $\psi = (180 \pm 0)°$, which corresponds to a pure left-handed chiral Néel domain wall structure, a DW width of $\Delta_\mathrm{DW} = (20 \pm 4)$ nm, and an out-of-plane surface magnetization $M_z t = (48.9 \pm 3.2)$ μ_B nm^{-2}, which corresponds to $M_\mathrm{s} = (53 \pm 4)$ kA m^{-1}. These values are consistent with the ones presented in Fig. 2 for TmIG(8.3 nm), supporting the finding of a stronger DMI in an all-oxide structure without the presence of a metallic heavy metal layer.

Supplementary References

1. Espinosa, G. P. Crystal chemical study of the rare-earth iron garnets. *J. Chem. Phys.* **37**, 2344–2347 (1962).
2. Kubota, M. *et al.* Systematic control of stress-induced anisotropy in pseudomorphic iron garnet thin films. *J. Magn. Magn. Mater.* **339**, 63–70 (2013).
3. Paoletti, A. *Physics of Magnetic Garnets*. (North-Holland Company, 1978).
4. Shao, Q. *et al.* Role of dimensional crossover on spin-orbit torque efficiency in magnetic insulator thin films. *Nat. Commun.* **9**, 3612 (2018).
5. Avci, C. O. *et al.* Interface-driven chiral magnetism and current-driven domain walls in insulating mangetic garnets. *Nat. Nanotechnol.* **14**, 561–566 (2019).
6. Kubota, M. *et al.* Stress-induced perpendicular magnetization in epitaxial iron garnet thin films. *Appl. Phys. Express* **5**, 103002 (2012).
7. Chen, Y.-T. *et al.* Theory of spin Hall magnetoresistance. *Phys. Rev. B* **87**, 144411 (2013).
8. Avci, C. O. *et al.* Current-induced switching in a magnetic insulator. *Nat. Mater.* **16**, 309–314 (2017).
9. Vélez, S. *et al.* Hanle Magnetoresistance in Thin Metal Films with Strong Spin-Orbit Coupling. *Phys. Rev. Lett.* **116**, 016603 (2016).
10. Tang, C. *et al.* Anomalous Hall hysteresis in $Tm_3Fe_5O_{12}$/Pt with strain-induced perpendicular magnetic anisotropy. *Phys. Rev. B* **94**, 140403 (2016).
11. Avci, C. O. *et al.* Fast switching and signature of efficient domain wall motion driven by spin-orbit torques in a perpendicular anisotropy magnetic insulator/Pt bilayer. *Appl. Phys. Lett.* **111**, 072406 (2017).
12. Quindeau, A. *et al.* $Tm_3Fe_5O_{12}$/Pt Heterostructures with Perpendicular Magnetic Anisotropy for Spintronic Applications. *Adv. Electron. Mater.* **3**, 1600376 (2017).
13. Sagasta, E. *et al.* Tuning the spin Hall effect of Pt from the moderately dirty to the superclean regime. *Phys. Rev. B* **94**, 060412 (2016).
14. Jia, X., Liu, K., Xia, K. & Bauer, G. E. W. Spin transfer torque on magnetic insulators. *Europhys. Lett.* **96**, 17005 (2011).
15. Althammer, M. *et al.* Quantitative study of the spin Hall magnetoresistance in ferromagnetic insulator/normal metal hybrids. *Phys. Rev. B* **87**, 224401 (2013).
16. Kosub, T. *et al.* Anomalous Hall-like transverse magnetoresistance in Au thin films on $Y_3Fe_5O_{12}$. *Appl. Phys. Lett.* **113**, 222409 (2018).
